# Bis-Amiridines as Acetylcholinesterase and Butyrylcholinesterase Inhibitors: *N*-Functionalization Determines the Multitarget Anti-Alzheimer’s Activity Profile

**DOI:** 10.3390/molecules27031060

**Published:** 2022-02-04

**Authors:** Galina F. Makhaeva, Nadezhda V. Kovaleva, Natalia P. Boltneva, Elena V. Rudakova, Sofya V. Lushchekina, Tatiana Yu. Astakhova, Igor V. Serkov, Alexey N. Proshin, Eugene V. Radchenko, Vladimir A. Palyulin, Jan Korabecny, Ondrej Soukup, Sergey O. Bachurin, Rudy J. Richardson

**Affiliations:** 1 Institute of Physiologically Active Compounds, Russian Academy of Sciences, 142432 Chernogolovka, Russia; gmakh@ipac.ac.ru (G.F.M.); kovalevanv@ipac.ac.ru (N.V.K.); boltneva@ipac.ac.ru (N.P.B.); rudakova@ipac.ac.ru (E.V.R.); sofya.lushchekina@gmail.com (S.V.L.); serkoviv@mail.ru (I.V.S.); proshin@ipac.ac.ru (A.N.P.); bachurin@ipac.ac.ru (S.O.B.); 2 Emanuel Institute of Biochemical Physics, Russian Academy of Sciences, 119334 Moscow, Russia; astakhova1967.t@yandex.ru; 3 Department of Chemistry, Lomonosov Moscow State University, 119991 Moscow, Russia; genie@qsar.chem.msu.ru (E.V.R.); vap@qsar.chem.msu.ru (V.A.P.); 4 Biomedical Research Centre, University Hospital Hradec Kralove, 500 05 Hradec Kralove, Czech Republic; jan.korabecny@fnhk.cz (J.K.); ondrej.soukup@fnhk.cz (O.S.); 5 Department of Environmental Health Sciences, University of Michigan, Ann Arbor, MI 48109, USA; 6 Department of Neurology, University of Michigan, Ann Arbor, MI 48109, USA; 7 Center of Computational Medicine and Bioinformatics, University of Michigan, Ann Arbor, MI 48109, USA; 8 Michigan Institute for Computational Discovery and Engineering, University of Michigan, Ann Arbor, MI 48109, USA

**Keywords:** amiridine, *N*-acylamide, thiourea, acetylcholinesterase (AChE), butyrylcholinesterase (BChE), antioxidants, β-amyloid (Aβ_42_), neuroprotection, ADMET, Alzheimer’s disease (AD)

## Abstract

Using two ways of functionalizing amiridine—acylation with chloroacetic acid chloride and reaction with thiophosgene—we have synthesized new homobivalent bis-amiridines joined by two different spacers—bis-*N*-acyl-alkylene (**3**) and bis-*N*-thiourea-alkylene (**5**) —as potential multifunctional agents for the treatment of Alzheimer’s disease (AD). All compounds exhibited high inhibitory activity against acetylcholinesterase (AChE) and butyrylcholinesterase (BChE) with selectivity for BChE. These new agents displayed negligible carboxylesterase inhibition, suggesting a probable lack of untoward drug–drug interactions arising from hydrolytic biotransformation. Compounds **3** with bis-*N*-acyl-alkylene spacers were more potent inhibitors of both cholinesterases compared to compounds **5** and the parent amiridine. The lead compounds **3a–c** exhibited an IC_50_(AChE) = 2.9–1.4 µM, IC_50_(BChE) = 0.13–0.067 µM, and 14–18% propidium displacement at 20 μM. Kinetic studies of compounds **3a** and **5d** indicated mixed-type reversible inhibition. Molecular docking revealed favorable poses in both catalytic and peripheral AChE sites. Propidium displacement from the peripheral site by the hybrids suggests their potential to hinder AChE-assisted Aβ_42_ aggregation. Conjugates **3** had no effect on Aβ_42_ self-aggregation, whereas compounds **5c**–**e** (*m* = 4, 5, 6) showed mild (13–17%) inhibition. The greatest difference between conjugates **3** and **5** was their antioxidant activity. Bis-amiridines **3** with *N*-acylalkylene spacers were nearly inactive in ABTS and FRAP tests, whereas compounds **5** with thiourea in the spacers demonstrated high antioxidant activity, especially in the ABTS test (TEAC = 1.2–2.1), in agreement with their significantly lower HOMO-LUMO gap values. Calculated ADMET parameters for all conjugates predicted favorable blood–brain barrier permeability and intestinal absorption, as well as a low propensity for cardiac toxicity. Thus, it was possible to obtain amiridine derivatives whose potencies against AChE and BChE equaled (**5**) or exceeded (**3**) that of the parent compound, amiridine. Overall, based on their expanded and balanced pharmacological profiles, conjugates **5c**–**e** appear promising for future optimization and development as multitarget anti-AD agents.

## 1. Introduction

Alzheimer’s disease (AD) consists of pronounced degenerative changes in the aging brain, including widespread synaptic dysfunction and neuronal loss, leading to profound deficits in memory and cognition [1]. At present, there is no way to prevent or ameliorate AD, and as the proportion of elderly people has increased in the global population, the impact of the disease has grown to epidemic proportions [2]. Despite decades of research attempting to delineate disease mechanisms at the molecular level, a clear understanding of these processes has remained elusive. However, it has become apparent that although age is the overriding determinant, not everyone who reaches an advanced age develops AD. Therefore, the risk of developing AD must be dependent upon one or more additional factors, i.e., the etiology is multifactorial, and investigators are still in the process of identifying these ancillary variables [3].

To date, the main successes have been the characterization of the pathogenic features of AD. These include neuronopathy (particularly of cholinergic neurons), synaptic dysfunction and loss leading to impaired neurotransmitter systems, mitochondrial dysfunction leading to oxidative stress, and the accumulation of misfolded or otherwise aberrant proteins such as tau and β-amyloid [3,4,5].

Among other processes that are important for cognitive functioning, cholinergic signaling undergoes a steady decline in AD, marked by a progressive decrease in the levels of the neurotransmitter, acetylcholine (ACh). A straightforward way to increase ACh concentrations is to suppress ACh degradation by inhibiting acetylcholinesterase (AChE) with anticholinesterase drugs. Those in current therapeutic use include rivastigmine (Exelon), galantamine (Reminyl), and donepezil (Aricept) [6,7].

Normally, brain ACh predominantly undergoes hydrolysis (80%) by AChE, and hydrolysis by butyrylcholinesterase (BChE) is supplementary. However, as AD progresses, AChE activity declines and BChE activity increases [8,9,10]. Recognition of this phenomenon has figuratively thrown a spotlight on BChE as a viable druggable target to help overcome the decline in brain ACh concentration that occurs in AD [11,12,13,14]. Consequently, the administration of agents that act against both AChE and BChE is thought to boost therapeutic efficiency [12,15]. Nevertheless, it should be borne in mind that anticholinesterases on their own, without possessing any disease-modifying effects, are only capable of alleviating symptoms in the short term; they are not able to halt the underlying disease processes [6,16].

A pathognomonic feature of AD is the deposition of β-amyloid peptide (Aβ) plaques in the brain [17]. This process, known as Aβ amyloidogenesis, is complex, involving a series of intermediate steps leading to the mature fibril [18]. However, it is also thought that soluble oligomers of Aβ constitute an important early stage in the pathogenic process [19,20,21]. Accordingly, compounds that inhibit Aβ aggregation could have an ameliorative, disease-modifying effect [22,23].

We now know that AChE can mediate functions that have a bearing on AD pathogenesis, namely, its peripheral anionic site (PAS) can bring about the aggregation of Aβ [24,25,26]. This finding has prompted a new research direction aimed at discovering drug candidates capable of both inhibiting AChE activity and its capacity to facilitate the formation of Aβ aggregates [27,28,29,30,31,32]. Agents possessing both these abilities could perform dual functions as cognition enhancers and disease modifiers [33,34,35]. BChE has also been shown to participate in one or more steps leading to Aβ aggregation [8,36,37]. Consequently, from the standpoints of cognition enhancement by elevating ACh levels as well as disease modification by blocking Aβ aggregation, it makes sense to search for compounds capable of inhibiting both AChE and BChE.

An important contributor to the initiation and continuation of neurodegenerative processes is oxidative stress, which occurs when the capacity of cellular antioxidant systems is insufficient to overcome the creation of reactive oxygen species (ROS) [38,39]. Oxidative stress can both promote and be fueled by pathologic states associated with neurodegeneration, such as the dysfunction of mitochondria, disruption of metal ion homeostasis, and formation and deposition of Aβ aggregates [40,41,42]. These pathogenic features of ROS justify the incorporation of antioxidants in AD therapeutics [40,42,43,44]. Accordingly, in the search for potential therapeutic agents for AD, it could be beneficial to discover anticholinesterase compounds that could perform a double duty as antioxidants [32,44,45,46,47,48].

There is considerable interest in the discovery and development of multi-target-directed ligands (MTDLs), also known as multifunctional molecules or multitarget drugs or agents [4,40,49,50]. This interest has arisen from the realization that complex diseases such as AD involve dysfunctions in multiple processes and targets, so that the former approach of searching for a single druggable target for a given disease is unlikely to be successful in this case [51,52,53,54,55].

The design of multitarget agents is a rational approach that typically involves splicing two distinct pharmacophores separated by a spacer into a single molecule. Alternatively, two identical pharmacophores could be used to produce homobivalent ligands or homodimers. As a starting point, individual pharmacophores should be used that are known to exert an action on a given target [56,57,58]. This splicing of pharmacophores can result in emergent properties that increase the pharmacologic activity of one or both pharmacophores or bring about new activities that are not seen in either of the component pharmacophores [21,22,49,59,60,61,62].

A typical example in the search for component pharmacophores for use in anti-AD MTDLs is tacrine, a molecule that is widely used for developing both heterodimers and homodimers, exhibiting a broad pharmacological profile [21,22,30,47,63,64,65,66,67,68]. Furthermore, an illustrative example of a homodimer that proved to exhibit multitarget capabilities is the tacrine homodimer bis(7)-tacrine or bis(7)-cognitin, B7C. In this case, one of the tacrine fragments binds to the CAS AChE region, close to the enzyme catalytic triad, while the other tacrine component binds to the PAS at the entrance of the catalytic gorge. As a result, B7C is one of the most effective AChE inhibitors, as well as a potent blocker of AChE-induced β-amyloid aggregation. Additionally, B7C was found to be an inhibitor of β-amyloid self-aggregation, a noncompetitive antagonist of NMDA receptors that can prevent glutamate-induced damage to hippocampal neurons, an antagonist of a GABA_A_ receptor, and an inhibitor of the nitric oxide synthase signaling pathway [69]. Thus, in contrast to its parent compound tacrine, B7C is a multi-target compound that shows promising biological activity.

To create new homobivalent MTDLs within the present study, we used another anticholinesterase pharmacophore—the amiridine molecule. Amiridine is an anticholinesterase drug that has found use in Russia and some other countries in Eastern Europe as a therapeutic agent for AD and certain other neurological disorders [70,71,72,73,74,75]. Known to inhibit AChE and BChE, with more potency against the latter enzyme, amiridine also exhibits numerous other biological activities [76,77] that have been reviewed in our previous publication [78].

Amiridine includes a 4-aminopyridine fragment and resembles tacrine (9-amino-l,2,3,4-tetrahydroacridine). Nevertheless, unlike tacrine, amiridine lacks hepatotoxicity, exerts fewer CNS and PNS cholinergic side effects, and has a wider therapeutic window [76,79]. However, the amiridine structure has a lower reactivity compared to tacrine. In this regard, there is practically no information on amiridine derivatives in the literature, and the functionalization of the amiridine molecule at the external nitrogen, while maintaining the unique amiridine scaffold, is a separate issue [80,81].

Recently, our team performed the functionalization of the amiridine molecule by acylation with chloroacetic acid chloride. The resulting amiridine chloroacetamide was reacted with piperazines, resulting in the production of a number of amiridine-piperazine hybrids as prospective multifunctional agents for AD treatment [78]. In our current work, to obtain new amiridine-based MTDLs, we used the reaction of amiridine chloroacetamide with diaminoalkanes to synthesize amiridine–amiridine homodimers with bis-*N*-acyl-alkylene spacers. Furthermore, we developed an additional way of functionalizing amiridine with thiophosgene, which enabled us to obtain amiridine homodimers with bis-N-thiourea-alkylene spacers.

**Figure 1:**
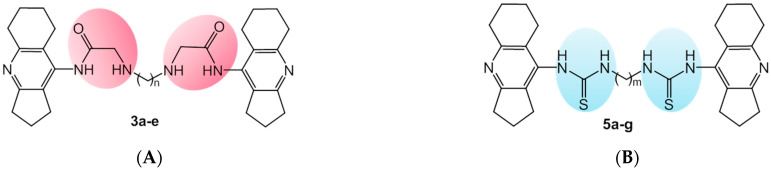
Structures of bis-amiridines: (**A**) **3**, highlighting the bis-*N*-acyl-alkylene spacers; (**B**) **5**, highlighting the bis-*N*-thiourea-alkylene spacers.

Here, we present the synthesis and comparative investigation of the pharmacological characteristics of new homobivalent ligands of bis-amiridine series **3** and **5** (Figure 1), obtained as a result of two different methods of amiridine functionalization, as potential MTDLs for AD treatment. Our work comprised measurement of the esterase profile of the conjugates, i.e., their inhibitory activity against AChE, BChE, and a structurally related enzyme, carboxylesterase (CES, EC 3.1.1.1), along with the determination of mechanistic insights into their esterase inhibition via enzyme kinetics and quantum mechanics (QM)-assisted molecular docking. In addition, we measured propidium iodide displacement from the AChE PAS to assess the potential of the conjugates as blockers of AChE-induced Aβ aggregation, and determined the ability of the compounds to inhibit β-amyloid (1–42) (Aβ_42_) self-aggregation. To evaluate the antioxidant activity of the compounds, we carried out ABTS and FRAP assays along with a computer assessment of their HOMO-LUMO gap energies. Finally, to predict potential pharmacokinetic properties of the new structures, we computationally estimated their ADMET profiles.

## 2. Results and Discussion

### 2.1. Chemistry

Functionalization of the amiridine molecule and the preparation of amiridine derivatives are associated with a number of difficulties due to the structure of the parent molecule. For example, only a few amiridine derivatives were described in the literature (excluding patents), in contrast to tacrine. Whereas “9-Cl-tacrine” is mainly used for the synthesis of tacrine derivatives, the analogous “Cl-amiridine” is not easy to obtain; moreover, the chlorine in “Cl-amiridine” proved to be difficult to displace. The reduced activity of amiridine in reactions that are common for tacrine can be associated with both the steric factor of two polymethylene rings and the close pK_a_ values of the endo- and exocyclic nitrogen atoms [80,81].

**Scheme 1:**
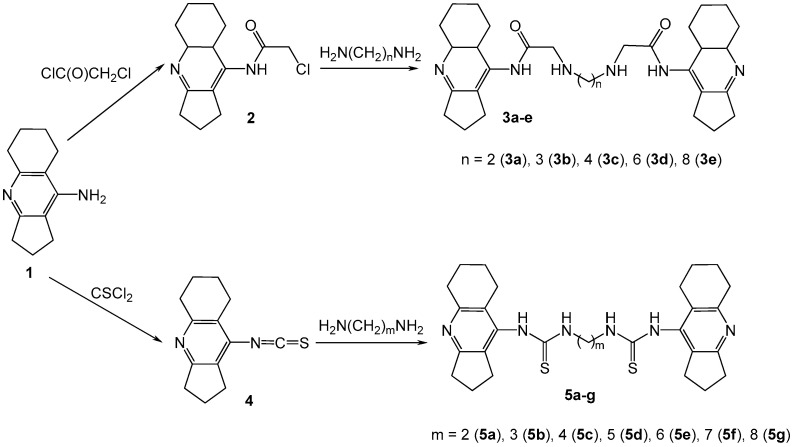
Synthesis of bis-amiridines **3** and **5**.

As is known, similarly to 4-aminopyridine, the alkylation reactions of amiridine proceed at the endocyclic nitrogen atom, and the acylation reactions proceed at the exocyclic amino group nitrogen atom [80]. Due to this, we started the functionalization of the amiridine molecule at the external nitrogen by acylation with chloroacetic acid chloride. The obtained amiridine chloroacetamide was reacted with piperazines, providing amiridine-piperazine conjugates [78]. In this work, we used the reaction of amiridine chloroacetamide **2** with 1,ω-diaminoalkanes to prepare bis-amiridines **3** with bis-*N*-acyl-alkylene spacers (Scheme 1). The reaction was performed in DMF in the presence of K_2_CO_3_ as a base.

The second method we used for the functionalization of the amiridine molecule was to react the external nitrogen with thiophosgene to obtain the corresponding isothiocyanate **4** (Scheme 1). The synthesis was carried out heterogeneously in a chloroform–water mixture in the presence of NaHCO_3_. The progress of the amine conversion reaction (5–7 h) was monitored by NMR. This approach allowed us to obtain amiridine isothiocyanate **4**, although, thus far, with a low yield. Attempts are being made to improve the technique to increase the yield.

By reacting amiridine isothiocyanate **4** with 1,ω-diaminoalkanes in chloroform at room temperature, we obtained a series of bis-amiridines **5** joined through bis-*N*-thiourea-alkylene spacers (Scheme 1).

We characterized all of the synthesized compounds by ^1^H spectroscopy and elemental analysis. We also characterized ten of the synthesized compounds by ^13^C NMR spectroscopy. Thus, we developed methods for the synthesis of bis-amiridines **3** and **5** (Scheme 1) with two different spacers, and we prepared the compounds for biological studies.

### 2.2. Inhibition Studies of AChE, BChE and CES. Structure-Activity Relationships

By assessing the esterase profile of new potential anti-AD compounds, we enabled an estimation of their primary pharmacological effects—AChE and BChE inhibition—and possible untoward side effects—CES inhibition, which would block an important route of hydrolytic biotransformation of numerous ester-containing drugs [82,83,84]. For our evaluation of the esterase profile, we used human erythrocyte AChE, equine serum BChE, and porcine liver CES. As in our previous studies, we used these non-human sources of BChE and CES in consideration of their relatively low cost and high sequence identity to the corresponding human enzymes [83,85,86], as well as the exploratory nature of our investigation.

We characterized the inhibitory activities of the test compounds against the esterases either as the percent inhibition achieved at an inhibitor concentration of 20 µM or as the IC_50_—the inhibitor concentration required to decrease a given enzyme activity by 50%. Positive controls were tacrine, a known AChE and BChE inhibitor, and bis-4-nitrophenyl phosphate (BNPP), a selective CES inhibitor. The results are displayed in Table 1.

The study of the esterase profile of the synthesized bis-amiridines showed (Table 1) that all conjugates exhibit a rather high inhibitory activity against both cholinesterases. Like amiridine, they are more effective against BChE and very weakly inhibit CES.

Compounds **3** with a bis-*N*-acyl-alkylene spacer and *n* = 2–6 exhibit a high inhibitory activity against AChE that exceeds the activity of the parent amiridine by 1.5–3 times, while there is no noticeable dependence of the activity on the number of methylene units in the spacer. The activity decreases by an order of magnitude with an increase in spacer up to *n* = 8 (compound **3e**).

As for compounds **5** with bis-thiourea-alkylene spacers, an increase in the spacer length from *m* = 2 (**5a**) to *m* = 5 (**5d**) led to a 24-fold enhancement of anti-AChE activity. The most active compounds against AChE were conjugates **5d** and **5e** with *m* = 5 and 6, respectively. Their inhibitory activity is equal to that of the parent compound amiridine. Further spacer elongation results in a decrease in anti-AChE activity.

With regard to BChE inhibition, compounds **3a**–**c** with bis-*N*-acyl-alkylene spacers are 3–4 times more potent inhibitors than the parent compound amiridine. The most active is compound **3c** (*n* = 4): IC_50_ = 0.067 ± 0.001 μM. As for compounds **5** with bis-thiourea-alkylene spacers, their anti-BChE activity is decreased for compounds with short (*m* = 2) and long (*m* = 7, 8) spacers, while compounds **5b**–**e** (*m* = 3–6) are more active and demonstrate rather close anti-BChE activity in the submicromolar region with IC_50_ = 0.7–0.8 μM.

In general, compounds **3** with bis-*N*-acyl-alkylene spacers are more potent inhibitors of both cholinesterases compared to compounds **5** with bis-thiourea-alkylene spacers. In both classes of bis-amiridines, **3** and **5**, compounds with a long spacer (*n*/*m* = 8) have lower activity.

Thus, for the first time, it was possible to obtain amiridine derivatives exceeding the parent amiridine in their ability to inhibit AChE and BChE.

### 2.3. Kinetic Studies of AChE and BChE Inhibition

**Figure 2:**
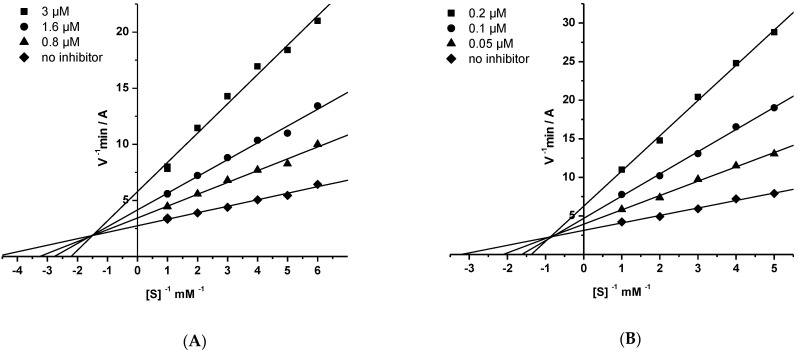

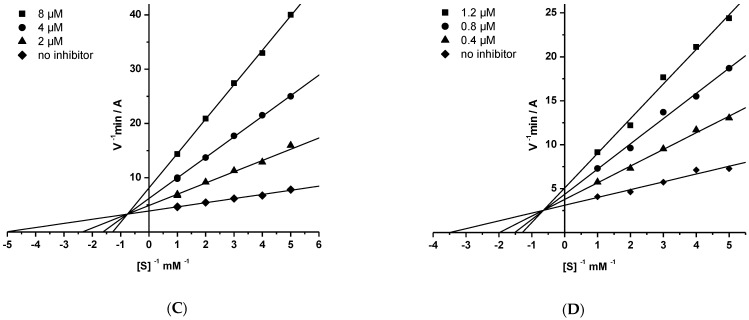
Steady state inhibition of (**A**) AChE and (**B**) BChE by compound **3a**; Steady state inhibition of (**C**) AChE and (**D**) BChE by compound **5d**.

We carried out investigations of the kinetics and mechanism of AChE and BChE inhibition by the synthesized conjugates using compounds from groups **3a** and **5d**. Graphical analysis of the kinetic data on AChE (Figure 2A,C) and BChE (Figure 2B,D) inhibition by compounds **3a** and **5d**, respectively, from the Lineweaver–Burk double-reciprocal plots, showed changes in both K_m_ and V_max_, suggesting mixed-type inhibition.

Inhibition constants of compound **3a** for AChE were 0.772 ± 0.047 µM (*K*_i_, the competitive component) and 2.62 ± 0.13 µM (α*K*_i_, the non-competitive component). Corresponding values for BChE were 0.0578 ± 0.0025 µM (*K*_i_) and 0.189 ± 0.007 µM (α*K*_i_). Inhibition constants of compound **5d** for AChE were 1.18 ± 0.08 µM (*K*_i_) and 7.75 ± 0.11 µM (α*K*_i_). Corresponding values for BChE were 0.340 ± 0.012 µM (*K*_i_) and 1.85 ± 0.06 µM (α*K*_i_).

### 2.4. Molecular Modeling Studies

#### 2.4.1. Characterization of the Compounds

For compounds **3,** the most stable was the *trans*-conformation of the amide bond, as expected. However, for compounds **5,** the most stable was the *anti*-conformation of the thiourea fragment.

p*K*_a_ values estimated with ChemAxon (See Supplementary, Tables S1 and S2) for the amiridine fragments of compounds **3** and **5** were close to 7.4, which leads to a high pH-sensitivity in the distribution of macrospecies. For compounds **3,** the secondary amine groups in the spacer tend to be protonated, although for compounds **3a** and **3b**, p*K*_a_ values were lower than for the others, which led to a higher diversity of co-existing protonated forms. The most populated protonated forms were taken for further study using molecular docking.

#### 2.4.2. Molecular Docking

**Figure 3:**
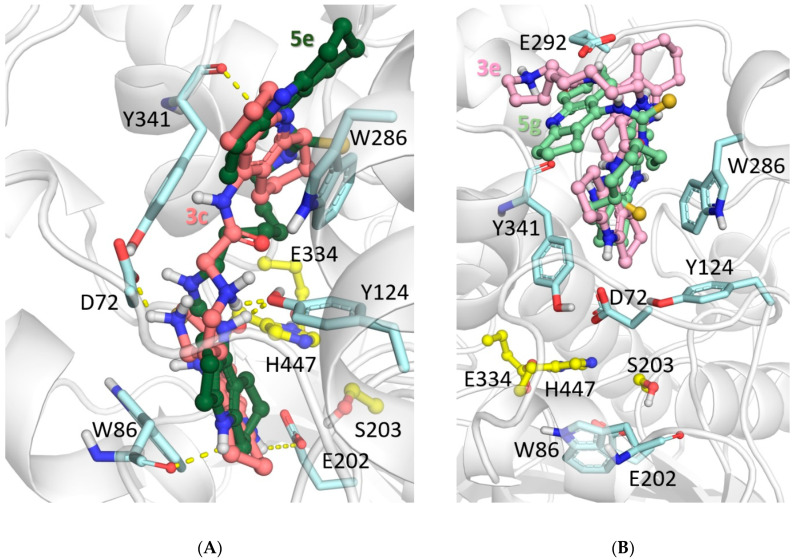
Molecular docking of compounds **3** (carbon atoms are shown in shades of red) and **5** (carbon atoms are shown in shades of green) into the AChE active site. (**A**) Comparative binding of compounds **3c** and **5e**. (**B**) Binding of compounds **3e** and **5g** to the PAS.

Molecular docking of the inhibitors into the active site of human AChE showed that, depending on the linker structure and interactions of its functional groups with the gorge residues, compounds of series **3** and **5** have slightly different positions in the CAS. For compounds **3**, a positively charged amiridine group interacts with the Glu202 side chain, and for compounds **5,** it forms a hydrogen bond with the Trp86 main chain oxygen atom (Figure 3A). In the case of compounds **5**, the exocyclic amino group of the amiridine fragment forms a hydrogen bond with the Tyr124 side chain, while for compounds **3**, one of the positively charged amino groups interacts with this residue, allowing the amiridine fragment to enter deeper into the gorge. We had previously observed such a difference in the position of the pharmacophore depending on the linker structure for tacrine derivatives [30,87], and previous molecular dynamics simulations of amiridine derivatives showed that such interactions are rather stable [78]. Otherwise, based on the best binding pose, homological compounds **3a**–**3e** bind to the active site and the gorge similarly, regardless of the length of the spacer (Figure S26A). This pattern of binding can be attributed to the interactions of the positively charged secondary amino group with aromatic and carboxylic residues, such as Trp286, Tyr341, and Asp74, along with the high flexibility of the bis-*N*-acyl-alkylene spacers. Compounds **5a**–**g**, with more rigid thiourea-based spacers, bind in a uniform way, with one amiridine group adjacent to the active site and the second amiridine group in the PAS, forming a hydrogen bond between the NH-group and the Tyr341 main chain oxygen atom. With linker extension, compounds protrude into the PAS more prominently, although the conjugates with the longest tethers (**5e**–**5g**) do not show notable differences in binding poses (Figure S26B).

Among all docked binding poses, there are several solely belonging to the PAS (Figure 3B). In this case, the compounds from series **3** show about 2 kcal/mol better binding affinity, which could explain their improved displacement of propidium from the PAS relative to series **5** compounds.

**Figure 4:**
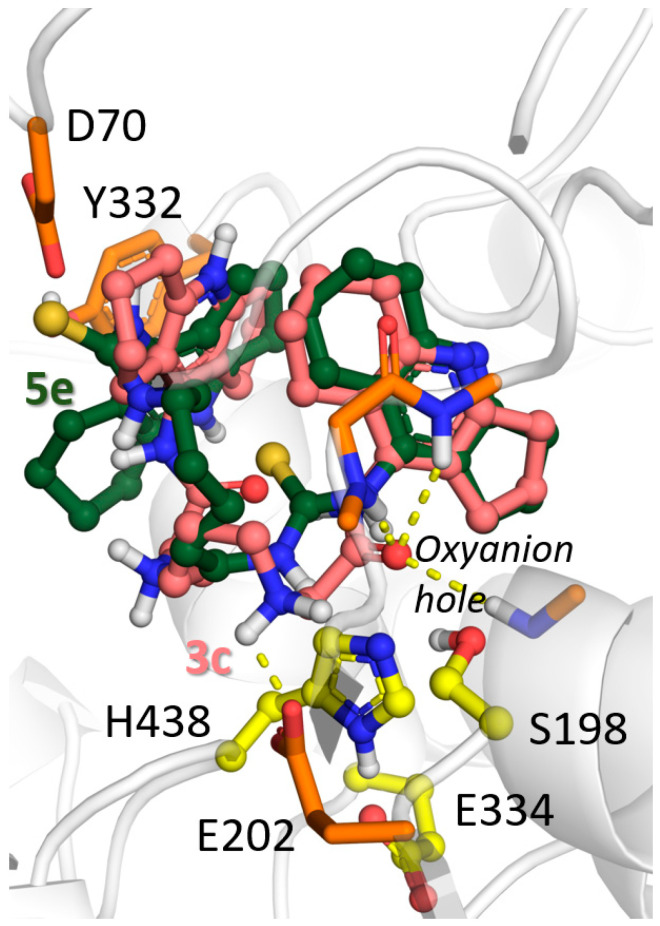
Molecular docking of compounds **3c** (carbon atoms are shown pink) and **5e** (carbon atoms are shown green) into the BChE active site.

With respect to binding to BChE, the bis-*N*-acyl-alkylene spacers of compounds **3** exerted the following binding pattern: contact of the acyl-group with the oxyanion hole and ionic interactions of the protonated secondary amino group with Glu202 (Figure 4). This binding makes the position of the amiridine groups in the CAS very stable, while the second amiridine moiety protrudes into the PAS (Figure S27A). In contrast, compounds **5** form no specific interactions. However, their uniform binding to the active site gorge consistently indicates that, regardless of the spacer length (Figure S27B), hydrophobic non-specific interactions are strong enough to ensure a good binding. Considering the relative rigidness of the spacer, the steric strain would be expected to grow with increasing spacer length.

### 2.5. Displacement of Propidium Iodide from the PAS of EeAChE

Considering the ability of the AChE peripheral anionic site (PAS) to induce β-amyloid aggregation [24,25,26], we evaluated conjugates **3** and **5** for their ability to displace propidium iodide (a selective PAS ligand) from the *Ee*AChE PAS. This method is widely used to screen compounds for their potential ability to block AChE-induced β-amyloid aggregation. This is because when compounds bind to the AChE PAS, they prevent β-amyloid binding, thereby inhibiting its AChE-facilitated aggregation [30,88,89,90,91,92]. As a positive control and reference compound, we used donepezil, a mixed-type AChE inhibitor for which the ability to block AChE-PAS-induced Aβ aggregation has been demonstrated [88]. The results are presented in Table 1.

It can be seen (Table 1) that conjugates **3** with bis-*N*-acyl-alkylene spacer were able to bind to the PAS of *Ee*AChE and displace propidium iodide either at similar levels (11–13%) to that of the reference compound donepezil (11.9 ± 0.9%), or even more effectively (15–18%). Compounds **3a**–**c** with spacer length *n* = 2, 3, 4 more effectively displaced propidium iodide from the AChE PAS than the parent amiridine molecule (12.2 ± 0.9%). Compounds **5a**–**g** with bis-thiourea-alkylene spacers were somewhat less effective (2–11%) than their analogs **3a**–**e**.

Taken together, the results from propidium iodide displacement, kinetics, and molecular docking suggest that conjugates **3** and **5** are AChE inhibitors that bind to the PAS of AChE, thereby exhibiting the potential to suppress the AChE-induced aggregation of β-amyloid.

### 2.6. Inhibition of β-Amyloid(1–42) (Aβ_42_) Self-Aggregation

The inhibitory activity of conjugates **3** and **5** against the self-aggregation of Aβ_42_ was determined *in vitro* by using a Thioflavin T (ThT)-based fluorimetric assay [88,93]. According to [94], for an initial high-throughput screening approach, the ThT-based assay is a well-suited quantitative technique because it affords the screening of a high number of new molecular entities and the selection of active compounds. The selectivity of ThT binding for fibrils suggests that active compounds resulting from this assay interfere with fibril formation without any information on the amyloid species targeted by the inhibitor.

The results reported in Table 1 demonstrate that the conjugates **3** and **5** had different effects on Aβ_42_ self-aggregation. Compounds **3a**–**e** containing a bis-*N*-acyl-alkylene spacer did not inhibit Aβ_42_ self-aggregation at all, whereas the Aβ_42_ anti-aggregation activity of the conjugates **5** depended on the length of the alkylene spacer. The conjugates **5a**,**b** with spacer length *m* = 2, 3 and the conjugates **5f**,**g** (*m* = 7, 8) weakly inhibit Aβ_42_ self-aggregation. Compounds **5c**–**e** (*m* = 4, 5, 6) showed percent inhibition values in the range of 12–17%. They were rather weak inhibitors of Aβ_42_ self-aggregation compared to the reference compounds Myricetin and Propidium iodide but approximately twice exceeded the anti-aggregation activity of the parent pharmacophore amiridine, which was able to very weakly interfere (6.4 ± 0.5%) with amyloid aggregation.

### 2.7. Antioxidant Activity

We determined the primary antioxidant activity of conjugates **3**, **5** spectrophotometrically using both the ABTS and FRAP procedures. In addition, the ABTS assay is based on both hydrogen atom transfer (HAT) and single electron transfer (SET) mechanisms and evaluates the binding of a model ABTS radical cation (2,2′-azino-bis-(3-ethylbenzothiazoline-6-sulfonate), ABTS**^•+^**). The ferric-reducing antioxidant power (FRAP) assay involves an assessment of the iron-reducing activity of a compound, which occurs exclusively by the SET mechanism. The results are presented in Table 2.

As can be seen from Table 2, conjugates **3** with bis-*N*-acyl-alkylene spacers generally demonstrated very weak radical-scavenging activity and iron-reducing capacity, or did not possess these activities at all.

The combination of amiridine molecules using spacers with a thiourea fragment markedly changed the picture of enhancing the antioxidant capabilities of the synthesized conjugates. The results showed that all conjugates **5** with bis-thiourea-alkylene spacers, in contrast to conjugates **3**, exhibit a high radical-scavenging activity in the ABTS test, which exceeds that of Trolox (TEAC = 1.2–2.1). Compounds **5a** (*m* = 2) and **5b** (*m* = 3) are the most active. Moreover, all conjugates **5** proved to be rather fast scavengers of the ABTS^•+^ radical, demonstrating quite high rates of initial reaction: the maximum binding of the ABTS^•+^ radical at a concentration of 20 μM (equal to the IC_50_ of Trolox) was observed within 3–10 min.

Our results are in good agreement with data reported in the literature on the high antioxidant potential of the thiourea unit and its potential applicability in multitarget molecules by imparting antioxidant properties to them [95,96]. Recent studies also show that thiourea derivatives have an excellent ability to capture free radicals and act as antioxidants, as evidenced by their powerful scavenging of superoxide radical anions (O_2_^•–^) and hydroxyl radicals (OH^•^) [97,98,99].

In the FRAP assay, an improvement in the iron-reducing ability of compounds was also observed when the bis-*N*-acyl-alkylene spacer (conjugates **3**) was replaced by a bis-thiourea-alkylene moiety (conjugates **5**). In contrast to bis-amiridines **3**, compounds **5** with bis-thiourea-alkylene spacers demonstrate rather good iron-reducing activity (TE = 0.35–0.85). The highest activity is observed for compound **5g** with the maximum spacer length (*m* = 8).

Thus, in contrast to bis-amiridines **3** with *N*-acyl-alkylene spacers, compounds **5** with thiourea in the spacer demonstrate high antioxidant activity. This corresponds well to our calculated values for HOMO-LUMO gaps (Table 2). Lower gap values for compounds **5** reflect their higher antioxidant potential.

### 2.8. Prediction of ADMET, Physicochemical, and PAINS Profiles

The results of our computational estimates of selected ADMET and physicochemical properties for compounds **3** and **5** are shown in Table 3. All of the compounds had high or moderate predicted values for intestinal absorption, enabling their oral administration. Moreover, we could expect reasonable CNS activity in view of the moderate predicted blood–brain barrier permeability (brain concentration is about 3–10% of the plasma concentration), although some optimization of this parameter might be desirable. The cardiac toxicity risk parameters (hERG p*K_i_* and pIC_50_) fell within 3.6–5.8 log units for all the analyzed compounds, which was within the lower part of their possible range (3–9 log units). According to the commonly accepted drug-likeness guidelines, the predicted lipophilicities and aqueous solubilities, as well as the molecular weights of the compounds, were within or close to the desirable range for potential drug compounds although the molecular weights and, in some cases, LogP values, violated the original Rule-of-5 limits. In any event, noting that the compounds were outside of the model applicability domain, the predicted values were not fully reliable. The integral quantitative estimates of drug-likeness (QED) were in the 0.2–0.7 range. The Pan Assay INterference compoundS (PAINS) filter did not identify any structural alerts. Consequently, the predicted ADMET, physicochemical, and PAINS properties of the compounds were acceptable for potential lead compounds in the discovery phase. Nevertheless, additional studies and structure optimization would be desirable to help maximize safety and improve the pharmacokinetic profile.

## 3. Materials and Methods

### 3.1. Chemistry

All solvents, chemicals, and reagents were commercially obtained and used without additional purification. ^1^H and ^13^C NMR spectra were recorded on a DPX-200 NMR spectrometer (Bruker, Karlsruhe, Germany) using tetramethylsilane as an internal standard. Chemical shifts, δ, are given in parts per million (ppm), and spin multiplicities are given as s (singlet), br s (broad singlet), d (doublet), t (triplet), q (quartet) or m (multiplet). Coupling constants, *J*, are expressed in hertz (Hz). Melting points were recorded on a Stuart SMP10 Melting Point Apparatus (Stuart, Staffordshire, UK) and are uncorrected. Yields refer to isolated pure products and were not maximized. CHN analysis was performed on the ER-20 analyzer (Carlo-Erba, Val-de-Reuil, France). All compounds exhibited analytical and spectroscopic data that strongly agreed with their expected structures.

### 3.2. Synthesis of Compounds

The synthesis and characteristics of compounds are shown below. All the synthesized compounds were characterized by ^1^H NMR spectroscopy and elemental analysis, and ten of the compounds were also characterized by ^13^C spectroscopy. The original NMR spectra are presented in Supplementary Materials (Figures S1–S23).

#### 3.2.1. Synthesis of Compounds **3a**–**e**

Bis-amiridines **3** with bis-*N*-acyl-alkylene spacers were synthesized by the reaction in DMF of 1,ω-diaminoalkanes with amiridine chloroacetamide **2**. Amiridine chloroacetamide **2** was synthesized by us earlier by the reaction of amiridine **1** with chloroacetic acid chloride [78].

1,ω-Diaminoalkane (1 mmol) and K_2_CO_3_ (3 mmol) were added, under mechanical stirring, to a solution of amiridine chloroacetamide **2** (2 mmol) in DMF (3 mL). The reaction mixture was heated at 60 °C for 3 h. After cooling, the mixture was poured into H_2_O (50 mL), extracted with CH_2_Cl_2_ (2 × 50 mL), washed with brine and dried. The solvent was removed in vacuo, and the residue was chromatographed on a silica gel column using methylene chloride/methanol (9/1) as eluent. The product was obtained as a white solid. Yield 32–45%.

*2,2′-[1,2-Ethanediylbis(imino)]bis[N-(2,3,5,6,7,8-hexahydro-1H-cyclopenta[b]quinolin-9-yl)-acetamide* (**3a**). White solid; Yield 44%, m.p. 184–186 °C. ^1^H NMR (CD_3_OD) δ: 1.62–1.98 (m, 8H, 2xCH_2_CH_2_), 2.02–2.25 (m, 4H, 2xCH_2_), 2.53–2.71 (m, 4H, 2xCH_2_), 2.75–3.05 (m, 12H, 6xCH_2_), 3.60–3.85 (m, 4H, NCH_2_CH_2_N). ^13^C NMR (CDCl_3_): 22.62, 22.84, 23.21, 24.48, 29.93, 32.27, 34.14, 45.24, 127.16, 132.69, 140.20, 156.96, 164.25, 181.03 (C=S). Anal. Calcd. for C_30_H_40_N_6_O_2_: C, 69.74; H, 7.80; N, 16.27. Found: C, 69.96; H, 7.61; N, 16.45.

*2,2′-[1,3-Propanediylbis(imino)]bis[N-(2,3,5,6,7,8-hexahydro-1H-cyclopenta[b]quinolin-9-yl)-acetamide* (**3b**). White solid; Yield 48%, m.p. 188–190 °C. ^1^H NMR (CDCl_3_) δ: 1.40–1.95 (m, 10H, 5xCH_2_), 1.97–2.21 (m, 4H, 2xCH_2_), 2.53–2.71 (m, 4H, 2xCH_2_), 2.75–3.05 (m, 12H, 6xCH_2_), 3.60–3.85 (m, 4H, 2xNCH_2_), 6.62 (br.s., 2H,2xNH), 7.68 (br.s., 2H,2xNH-Py). ^13^C NMR (CDCl_3_ +CD_3_OD): 22.57, 22.82, 23.14, 24.46, 29.34, 29.48, 32.23, 34.10, 41.36, 114.24, 119.22, 125.70, 127.27, 132.81, 140.72, 156.84, 164.03, 180.58 (C=S). Anal. Calcd. for C_31_H_42_N_6_O_2_: C, 70.16; H, 7.98; N, 15.84. Found: C, 70.34; H, 8.14; N, 15.67.

*2,2′-[1,3-Butanediylbis(imino)]bis[N-(2,3,5,6,7,8-hexahydro-1H-cyclopenta[b]quinolin-9-yl)-acetamide* (**3c**). White solid; Yield 40%, m.p. 192–194 °C. ^1^H NMR (CDCl_3_) δ: 1.40–1.70 (m, 4H, 2xCH_2_), 1.71–1.97 (m, 8H, 2xCH_2_CH_2_), 2.00–2.21 (m, 4H, 2xCH_2_), 2.53–2.71 (m, 4H, 2xCH_2_), 2.75–3.05 (m, 12H, 6xCH_2_), 3.50–3.73 (m, 4H, 2xNCH_2_), 6.62 (br.s., 2H,2xNH), 7.68 (br.s., 2H, 2xNH-Py). ^13^C NMR (CDCl_3_ +CD_3_OD): 22.76, 22.98, 23.30, 24.61, 26.64, 29.60, 32.30, 34.20, 44.70, 127.49, 133.16, 141.69, 156.64, 163.90, 180.86 (C=S). Anal. Calcd. for C_32_H_44_N_6_O_2_: C, 70.56; H, 8.14; N, 15.43. Found: C, 70.70; H, 8.00; N, 15.72.

*2,2′-[1,3-Hexanediylbis(imino)]bis[N-(2,3,5,6,7,8-hexahydro-1H-cyclopenta[b]quinolin-9-yl)-acetamide* (**3d**). White solid; Yield 46%, m.p. 193–195 °C. ^1^H NMR (CDCl_3_) δ: 1.21–1.35 (m, 4H, 2xCH_2_), 1.55–1.71 (m, 4H, 2xCH_2_), 1.73–1.93 (m, 8H, 2xCH_2_CH_2_), 2.02–2.23 (m, 4H, 2xCH_2_), 2.55–2.70 (m, 4H, 2xCH_2_), 2.75–3.11 (m, 12H, 6xCH_2_), 3.55–3.71 (m, 4H, 2xNCH_2_), 5.90 (br.s., 2H,2xNH), 7.31 (br.s., 2H, 2xNH-Py). ^13^C NMR (CDCl_3_ +CD_3_OD): 22.36, 22.72, 22.35, 23.04, 24.44, 26.05, 29.18, 32.70, 34.44, 44.40, 126.06, 131.19, 138.48, 157.98, 164.83, 180.30 (C=S). Anal. Calcd. for C_34_H_48_N_6_O_2_: C, 71.30; H, 8.45; N, 14.67. Found: C, 71.54; H, 8.21; N, 14.49.

*2,2′-[1,3-Octanediylbis(imino)]bis[N-(2,3,5,6,7,8-hexahydro-1H-cyclopenta[b]quinolin-9-yl)-acetamide* (**3e**). White solid; Yield 40%, m.p. 199–201 °C. ^1^H NMR (CDCl_3_) δ: 1.13–1.421 (m, 8H, 4xCH_2_), 1.43–1.67 (m, 4H, 2xCH_2_), 1.68–1.95 (m, 8H, 2xCH_2_CH_2_), 2.02–2.23 (m, 4H, 2xCH_2_), 2.53–2.72 (m, 4H, 2xCH_2_), 2.75–3.17 (m, 12H, 6xCH_2_), 3.43–3.75 (m, 4H, 2xNCH_2_), 5.72 (br.s., 2H,2xNH), 7.62 (br.s., 2H, 2xNH-Py). ^13^C NMR (CDCl_3_ +CD_3_OD): 22.51, 22.75, 23.13, 24.41, 26.31, 29.37, 32.17, 34.07, 44.42, 126.98, 132.52, 140.70, 156.82, 164.04, 180.39 (C=S). Anal. Calcd. for C_36_H_52_N_6_O_2_: C, 71.96; H, 8.72; N, 13.99. Found: C, 72.14; H, 8.78; N, 14.19.

#### 3.2.2. Synthesis of Intermediate **4**

Amiridine isothiocyanate **4** was synthesized by treating amiridine with thiophosgene (1.1 equiv) in a mixture of chloroform and saturated aqueous solution of NaHCO_3_ (1:1, *v*/*v*) at 0 °C for 5–7 h. The increase in the reaction time is due to the fact that, after 2 h of reaction, the NMR spectrum showed the presence (20–30%) of the amino group of the starting amiridine in the reaction mixture. Then, the layers were separated and the organic layer was evaporated. The residue was extracted with ether (2 × 30 mL). The ether extract was evaporated to give the desired isothiocyanate, which was used in the next step without further purification. Yield 20–25%.

*9-Isothiocyanato-2,3,5,6,7,8-hexahydro-1H-cyclopenta[b]quinoline* (**4**). Brown solid; Yield 22%, m.p. 103–105 °C. ^1^H NMR (CDCl_3_) δ: 1.75–2.03 (m, 4H, CH_2_CH_2_), 2.12–2.51 (m, 2H, CH_2_), 2.80 (br.s., 2H, CH_2_), 3.10 (t, 2H, J = 7.45 Hz, CH_2_), 3.21–3.36 (m, 2H, CH_2_), 3.10 (t, 2H, J = 7.71 Hz, CH_2_). ^13^C NMR (CDCl_3_): 21.40, 24.17, 25.16, 27.18, 31.27, 32.46, 35.42, 129.60, 135.98, 141.28, 144.48 (C=S), 156.96, 163.57. Anal. Calcd. For C_13_H_14_N_2_S: C, 67.79; H, 6.13; N, 12.16. Found: C, 67.54; H, 6.013; N, 12.26.

#### 3.2.3. Synthesis of Compounds **5a**–**g**

1,ω-Diaminoalkane (1 mmol) in chloroform (10 mL) was added dropwise to a stirred solution of isothiocyanatoamiridine **4** (2.1 mmol) in chloroform (10 mL). The reaction mixture was stirred at room temperature for 1 h. The reaction mixture was evaporated, and 10 mL of ether was added. The formed precipitate was collected on filter, washed with ether and dried. Yield 60–83%.

*2,2′-Ethane-1,2-diylbis[N-(2,3,5,6,7,8-hexahydro-1H-cyclopenta[b]quinolin-9-yl)(thiourea)]* (**5a**). White solid; Yield 78%, m.p. > 200 °C. ^1^H NMR (CD_3_OD) δ: 1.62–1.98 (m, 8H, 2xCH_2_CH_2_), 2.02–2.25 (m, 4H, 2xCH_2_), 2.53–2.71 (m, 4H, 2xCH_2_), 2.75–3.05 (m, 12H, 6xCH_2_), 3.60–3.85 (m, 4H, NCH_2_CH_2_N). ^13^C NMR (CDCl_3_ +CD_3_OD): 22.62, 22.84, 23.21, 24.48, 29.93, 32.27, 34.14, 45.24, 127.16, 132.69, 140.20, 156.96, 164.25, 181.03 (C=S). Anal. Calcd. for C_28_H_36_N_6_S_2_: C, 64.58; H, 6.97; N, 16.14. Found: C, 64.88; H, 6.92; N, 16.03.

*2,2′-Propane-1,2-diylbis[N-(2,3,5,6,7,8-hexahydro-1H-cyclopenta[b]quinolin-9-yl)(thiourea)]* (**5b**). White solid; Yield 82%, m.p. 188–190 °C. ^1^H NMR (CDCl_3_) δ: 1.40–1.95 (m, 10H, 5xCH_2_), 1.97–2.21 (m, 4H, 2xCH_2_), 2.53–2.71 (m, 4H, 2xCH_2_), 2.75–3.05 (m, 12H, 6xCH_2_), 3.60–3.85 (m, 4H, 2xNCH_2_), 6.62 (br.s., 2H,2xNH), 7.68 (br.s., 2H,2xNH-Py). ^13^C NMR (CDCl_3_ +CD_3_OD): 22.57, 22.82, 23.14, 24.46, 29.34, 29.48, 32.23, 34.10, 41.36, 114.24, 119.22, 125.70, 127.27, 132.81, 140.72, 156.84, 164.03, 180.58 (C=S). Anal. Calcd. for C_29_H_38_N_6_S_2_: C, 65.13; H, 7.16; N, 15.71. Found: C, 65.16; H, 7.33; N, 15.56.

*2,2′-Butane-1,2-diylbis[N-(2,3,5,6,7,8-hexahydro-1H-cyclopenta[b]quinolin-9-yl)(thiourea)]* (**5c**). White solid; Yield 83%, m.p. 194–196 °C. ^1^H NMR (CDCl_3_ + CD_3_OD) δ: 1.40–1.70 (m, 4H, 2xCH_2_), 1.71–1.97 (m, 8H, 2xCH_2_CH_2_), 2.00–2.21 (m, 4H, 2xCH_2_), 2.53–2.71 (m, 4H, 2xCH_2_), 2.75–3.05 (m, 12H, 6xCH_2_), 3.50–3.73 (m, 4H, 2xNCH_2_), 6.62 (br.s., 2H,2xNH), 7.68 (br.s., 2H, 2xNH-Py). ^13^C NMR (CDCl_3_ +CD_3_OD): 22.76, 22.98, 23.30, 24.61, 26.64, 29.60, 32.30, 34.20, 44.70, 127.49, 133.16, 141.69, 156.64, 163.90, 180.86 (C=S). Anal. Calcd. for C_30_H_40_N_6_S_2_: C, 65.66; H, 7.16; N, 15.31. Found: C, 65.80; H, 7.01; N, 15.59.

*2,2′-Pentane-1,2-diylbis[N-(2,3,5,6,7,8-hexahydro-1H-cyclopenta[b]quinolin-9-yl)(thiourea)]* (**5d**). White solid; Yield 88%, m.p. 185–187 °C. ^1^H NMR (CDCl_3_ + CD_3_OD) δ: 1.21–1.38 (m, 2H, CH_2_), 1.55–1.73 (m, 4H, 2xCH_2_), 1.75–1.97 (m, 8H, 2xCH_2_CH_2_), 2.02–2.21 (m, 4H, 2xCH_2_), 2.59–2.74 (m, 4H, 2xCH_2_), 2.75–3.11 (m, 12H, 6xCH_2_), 3.40–3.71 (m, 4H, 2xNCH_2_). ^13^C NMR (CDCl_3_ +CD_3_OD): 23.43, 23.63, 23.88, 25.13, 25.24, 29.80, 30.17, 32.81, 34.67, 45.70, 128.53, 134.21, 143.30, 156.83, 164.16, 181.65 (C=S). Anal. Calcd. for C_31_H_42_N_6_S_2_: C, 66.15; H, 7.52; N, 14.93. Found: C, 66.46; H, 7.32; N, 15.08.

*2,2′-Hexane-1,2-diylbis[N-(2,3,5,6,7,8-hexahydro-1H-cyclopenta[b]quinolin-9-yl)(thiourea)]* (**5e**). White solid; Yield 84%, m.p. 193–195 °C. ^1^H NMR (CDCl_3_) δ: 1.21–1.35 (m, 4H, 2xCH_2_), 1.55–1.71 (m, 4H, 2xCH_2_), 1.73–1.93 (m, 8H, 2xCH_2_CH_2_), 2.02–2.23 (m, 4H, 2xCH_2_), 2.55–2.70 (m, 4H, 2xCH_2_), 2.75–3.11 (m, 12H, 6xCH_2_), 3.55–3.71 (m, 4H, 2xNCH_2_), 5.90 (br.s., 2H,2xNH), 7.31 (br.s., 2H, 2xNH-Py). ^13^C NMR (CDCl_3_ +CD_3_OD): 22.36, 22.72, 22.35, 23.04, 24.44, 26.05, 29.18, 32.70, 34.44, 44.40, 126.06, 131.19, 138.48, 157.98, 164.83, 180.30 (C=S). Anal. Calcd. for C_32_H_44_N_6_S_2_: C, 66.63; H, 7.69; N, 14.57. Found: C, 66.66; H, 7.92; N, 14.46.

*2,2′-Heptane-1,2-diylbis[N-(2,3,5,6,7,8-hexahydro-1H-cyclopenta[b]quinolin-9-yl)(thiourea)]* (**5f**). White solid; Yield 82%, m.p. 169–171 °C. ^1^H NMR (CDCl_3_) δ: 1.11–1.40 (m, 6H, 3xCH_2_), 1.43–1.67 (m, 4H, 2xCH_2_), 1.68–1.95 (m, 8H, 2xCH_2_CH_2_), 2.00–2.25 (m, 4H, 2xCH_2_), 2.55–2.73 (m, 4H, 2xCH_2_), 2.75–3.15 (m, 12H, 6xCH_2_), 3.40–3.71 (m, 4H, 2xNCH_2_), 5.63 (br.s., 2H,2xNH), 7.67 (br.s., 2H, 2xNH-Py). ^13^C NMR (CDCl_3_): 22.32, 22.69, 22.99, 24.40, 26.48, 28.53, 28.96, 29.11, 32.69, 34.41, 45.29, 126.13, 131.19, 138.52, 157.98, 164.94, 179.90 (C=S). Anal. Calcd. for C_33_H_46_N_6_S_2_: C, 67.08; H, 7.85; N, 14.22. Found: C, 66.91; H, 7.75; N, 14.40.

*2,2′-Octane-1,2-diylbis[N-(2,3,5,6,7,8-hexahydro-1H-cyclopenta[b]quinolin-9-yl)(thiourea)]* (**5g**). White solid; Yield 88%, m.p. 190–192 °C. ^1^H NMR (CDCl_3_) δ: 1.13–1.421 (m, 8H, 4xCH_2_), 1.43–1.67 (m, 4H, 2xCH_2_), 1.68–1.95 (m, 8H, 2xCH_2_CH_2_), 2.02–2.23 (m, 4H, 2xCH_2_), 2.53–2.72 (m, 4H, 2xCH_2_), 2.75–3.17 (m, 12H, 6xCH_2_), 3.43–3.75 (m, 4H, 2xNCH_2_), 5.72 (br.s., 2H,2xNH), 7.62 (br.s., 2H, 2xNH-Py). ^13^C NMR (CDCl_3_ +CD_3_OD): 22.51, 22.75, 23.13, 24.41, 26.31, 29.37, 32.17, 34.07, 44.42, 126.98, 132.52, 140.70, 156.82, 164.04, 180.39 (C=S). Anal. Calcd. for C_34_H_48_N_6_S_2_: C, 67.51; H, 8.00; N, 13.89. Found: C, 67.67; H, 8.18; N, 14.05.

### 3.3. Biological Assays

#### 3.3.1. Enzymatic Assays

##### *In Vitro* AChE, BChE, and CES Inhibition

All experiments were carried out in accordance with the standard protocols approved by IPAC RAS. The following items were purchased from Sigma-Aldrich (St. Louis, MO, USA): human erythrocyte AChE, equine serum BChE, porcine liver CES, acetylthiocholine iodide (ATCh), butyrylthiocholine iodide (BTCh), 5,5′-dithio-bis-(2-nitrobenzoic acid) (DTNB), 4-nitrophenol acetate (4-NPA), tacrine, and BNPP. We measured the activity of AChE and BChE according to the colorimetric Ellman procedure (λ = 412 nm), as described in detail in [87]. CES activity was assessed as described in [87] by following the release of 4-nitrophenol spectrophotometrically (λ = 405 nm) using 4-NPA as a substrate. Freshly prepared solutions of the enzymes were used, which retained a constant activity during the experiment (2–2.5 h). Chromophore absorbances were measured with a FLUOStar Optima microplate reader (BMG Labtech, Ortenberg, Germany). DMSO (2% *v*/*v*) was employed as the solvent; the concentration used did not alter the activities of the enzymes (data not shown). Initially, we used a single concentration of 20 µM for all compounds. Subsequently, IC_50_ values (µM) were determined for the most active compounds against AChE, BChE, and CES (Figures S24 and S25).

##### Kinetic Study of AChE and BChE Inhibition. Determination of Steady-State Inhibition Constants

We assessed the mechanisms of AChE and BChE inhibition by performing a thorough analysis of enzyme kinetics. After a 5 min incubation at 25 °C (for temperature equilibration) with three increasing concentrations of inhibitor and six decreasing substrate concentrations, the residual enzyme activity was measured as described above for enzymatic assays. Linear regression of 1/V versus 1/[S] double-reciprocal (Lineweaver-Burk) plots was used to determine the inhibition constants for the competitive component (*K*_i_) and noncompetitive component (α*K*_i_).

#### 3.3.2. Propidium Iodide Displacement Studies

We used the fluorescence method to detect the propensity of the test compounds to competitively displace propidium iodide (Sigma-Aldrich, St. Louis, MO, USA); a selective ligand of the AChE PAS) [100,101]. Donepezil and tacrine (Sigma-Aldrich) were employed as the positive controls (i.e., reference compounds). The enzyme was *Electric eel* AChE (*Ee*AChE type VI-S, lyophilized powder, Sigma-Aldrich). We selected this source of AChE for consistency with our other reports and because of the purity, specific activity, and lower cost compared to human AChE. Moreover, a 3D alignment of *Ee*AChE (PDB: 1C2O) and human AChE (PDB: 4EY7) using the MUSTANG procedure [102] in YASARA-Structure 18.4.24 for Windows [103] yielded close agreement between the two structures (RMSD 0.623 Å over 527 aligned residues and 88.6% sequence identity).

The assay is based on the high level of fluorescence intensity of propidium iodide bound with AChE decreasing in the presence of test compounds that competitively displace propidium iodide from the AChE PAS [25,27]. Specificially, *Ee*AChE (7 μM final concentration) is incubated with the test compound (20 μM in 1 mM Tris-HCl buffer pH 8.0, 25 °C, for 15 min). Propidium iodide (final concentration 8 μM) is then added for a further 15-min incubation and the fluorescence spectrum is taken (530 nm (excitation) and 600 nm (emission)). The same concentration of propidium iodide in the Tris-HCI buffer was used as the blank. Triplicate determinations were recorded from a FLUOStar Optima microplate reader and results calculated via the following equation:

% Displacement = 100 − (IF_AChE+ Propidium + inhibitor_/IF_AChE + Propidium_) × 100 (1)

where IF_AChE + Propidium_ = fluorescence intensity of propidium iodide associated with AChE in the absence of the test compound (taken as 100%), and IF_AChE + Propidium + inhibitor_ = fluorescence intensity of propidium iodide associated with AChE in the presence of the test compound.

#### 3.3.3. Inhibition of β-Amyloid (1–42) (Aβ_42_) Self-Aggregation

The inhibitory effect of the test compounds toward Aβ_42_ self-aggregation was determined using the Thioflavin T (ThT) fluorescence method [88,93] with minor modifications. This assay is based on a specific interaction between the fluorescent dye thioflavin T that binds to the β-sheets of assembled amyloid fibrils leading to a significant increase in fluorescence signal [104]. Therefore, the decrease in ThT fluorescence correlates with the activity of studied compounds to inhibit the formation of amyloid aggregates.

A total of 1 mg of lyophilized HFIP-pretreated Aβ_42_ from rPeptide (Watkinsville, GA, USA) was dissolved in DMSO to obtain a stable initial solution ([Aβ_42_] = 500 μM), then it was aliquoted and stored at −20 °C.

For the measurement of Aβ_42_ self-aggregation and amyloid fibril inhibition studies by the tested compounds, aliquots of 500 μM Aβ_42_ stock solution were diluted in 215 mM Na-phosphate buffer pH = 8.0 to a final concentration of 50 μM Aβ_42_. Then, the samples were incubated for 24 h at 37 °C without stirring in the absence (base level of Aβ_42_ self-aggregation, control) or presence of the test compounds. Myricetin and propidium iodide were used as references (positive controls). All compounds were used at a concentration of 100 µM. To quantify Aβ_42_ fibril formation, after incubation, 5 μM ThT in 50 mM glycine-NaOH buffer pH 8.5 was added to the solutions to a final concentration of 4 μM ThT and the fluorescence was measured at 440 nm (excitation) and 485 nm (emission). Analyses were performed with a FLUOStar Optima microplate reader (LabTech, Ortenberg, Germany). The blanks consisted of 215 mM Na-phosphate buffer, pH = 8.0, 10% (*v*/*v*) DMSO or test compounds, respectively. Each assay was run in triplicate. Results are presented as mean ± SEM calculated using GraphPad Prism version 6.05 for Windows (San Diego, CA, USA).

The inhibition (%) of Aβ_42_ self-aggregation by the test compounds was calculated using the following equation:

% inhibition = 100 − (IF_i_/IF_o_) × 100 (2)

where IF_i_ and IF_o_ are the fluorescence intensities obtained for Aβ_42_ in the presence and in the absence of inhibitor, respectively, after subtracting the fluorescence of respective blanks.

#### 3.3.4. Antioxidant Activity

##### ABTS Radical Cation Scavenging Activity Assay

Radical scavenging activity of the compounds was assessed using the ABTS radical cation (ABTS^•+^) decolorization assay [105] with modifications, described in detail in [30,32]. ABTS (2,2′-azino-bis-(3-ethylbenzothiazoline-6-sulfonic acid)) was purchased from Tokyo Chemical Industry Co., Ltd. (Tokyo, Japan), potassium persulfate (dipotassium peroxodisulfate), Trolox (6-hydroxy-2,5,7,8-tetramethylchroman-2-carboxylic acid), HPLC-grade ethanol, and DMSO were obtained from Sigma-Aldrich. All tested compounds were dissolved in DMSO.

The reaction was monitored for 1 h with an interval of 1–10 min. Data are given for 1 h of incubation of compounds with ABTS^•+^ (100 μM final concentration). The reduction in absorbance was measured spectrophotometrically at 734 nm using xMark UV/VIS microplate spectrophotometer (Bio-Rad, Hercules, CA, USA). Ethanol blanks were run in each assay. Values were obtained from three replicates of each sample and three independent experiments. Standard antioxidant Trolox was used as a reference compound. Trolox equivalent antioxidant capacity (TEAC) values were determined as the ratio between the slopes obtained from the linear correlation of the ABTS radical absorbance with the concentrations of tested compounds and Trolox. For the test compounds, we also determined the IC_50_ values (compound concentration required for 50% reduction in the ABTS radical).

FRAP

The ferric reducing antioxidant power (FRAP) assay proposed by Benzie and Strain [106,107] was modified to be performed in 96-well microplates, as described in detail in [32]. The FRAP reagent contained 2.5 mL of 10 mM TPTZ (2,4,6-tris(2-pyridyl)-s-triazine, Sigma-Aldrich) solution in 40 mM HCl, 2.5 mL of 20 mM FeCl_3_ (Sigma-Aldrich) in distilled water and 25 mL of 0.3 M acetate buffer (pH 3.6). Aliquots of 10 µL of the tested compound (compounds **3**, **5** or reference substance) dissolved in DMSO (0.5 mM) were placed in quadruplicate. Absorbance was measured at the wavelength of 593 nm after 60-min incubation at 37 °C. In each case, Trolox was used as a reference compound to obtain the standard curve and value was calculated with respect to the activity of Trolox and expressed as Trolox equivalents (TE)—the values calculated as the ratio of the concentrations of Trolox and the test compound, resulting in the same effect on ferric reducing activity.

### 3.4. Molecular Modeling Studies

#### 3.4.1. Preparation of the Molecules

Conformers of the inhibitors were generated using OMEGA 4.0.0.4: OpenEye Scientific Software, Santa Fe, NM. http://www.eyesopen.com [108] (accessed on 20 January 2022). Generated conformers were optimized using a DFT quantum chemistry method (B3LYP/6-31G*, GAMESS-US [109] software https://www.msg.chem.iastate.edu/gamess/ accessed on 20 January 2022), and structures with the lowest energies were used for p*K*_a_ estimations and molecular docking simulations. Estimations of p*K*_a_ values were performed using the Calculator Plugins of Marvin 21.14.0, ChemAxon (http://www.chemaxon.com accessed on 20 January 2022). For molecular docking, the optimized structures of the ligands were used with partial atomic charges derived from QM results according to the Löwdin scheme [110]. Frontier orbitals energies were calculated with the B3LYP/6-311++G** level of theory.

#### 3.4.2. Molecular Docking

X-ray structures of human AChE co-crystallized with donepezil (PDB: 4EY7) [111] and an optimized X-ray structure of human BChE (PDB: 1P0I) [112,113] were used for molecular docking. Molecular docking was performed with AutoDock 4.2.6 software [114] (https://autodock.scripps.edu/download-autodock4/ accessed on 20 Jan 2022). The grid box for docking included the entire active site gorge of AChE (22.5 Å × 22.5 Å × 22.5 Å grid box dimensions) and BChE (15 Å × 20.25 Å × 18 Å grid box dimensions) with a grid spacing of 0.375 Å. The main Lamarckian Genetic Algorithm (LGA) [115] parameters were 256 runs, 25 × 10^6^ evaluations, 27 × 10^4^ generations, and a population size of 3000. Figures were prepared with PyMOL (www.pymol.org accessed on 20 January 2022).

### 3.5. Prediction of ADMET, Physicochemical, and PAINS Profiles

Lipophilicity (LogP_ow_) and aqueous solubility (pS) were estimated by the ALogPS 3.0 neural network model implemented in the OCHEM platform [116]. Human intestinal absorption (HIA) [117], blood–brain barrier distribution/permeability (LogBB) [118,119], and hERG-mediated cardiac toxicity risk (channel affinity p*K_i_* and inhibitory activity pIC_50_) [120] were estimated using the integrated online service for the prediction of ADMET properties [121]. This service implements predictive QSAR models based on accurate and representative training sets, fragmental descriptors, and artificial neural networks. The quantitative estimate of drug-likeness (QED) values [122] were calculated and the Pan Assay INterference compoundS (PAINS) alerts were checked using RDKit version 2020.03.4 software [123].

### 3.6. Statistical Analyses

All tests were performed at least in triplicate in three independent experiments. Results are presented as mean ± SEM calculated using GraphPad Prism version 6.05 for Windows (San Diego, CA, USA). Plots, linear regressions, and IC_50_ values were determined using Origin 6.1 for Windows, OriginLab (Northampton, MA, USA).

## 4. Conclusions

In summary, we developed two ways to functionalize the amiridine molecule: by acylation with chloroacetic acid chloride and by reaction with thiophosgene. The reaction of obtained intermediates, amiridine chloroacetamide and amiridine isothiocyanate, with 1,ω-diaminoalkanes allowed us to prepare bis-amiridines joined by two different spacers: bis-*N*-acyl-alkylene (**3**) and bis-*N*-thiourea-alkylene (**5**).

Our comparative studies of the pharmacological profiles of the new homobivalent ligands of series **3** and **5** as potential anti-AD agents allowed us to draw several conclusions regarding the influence of the spacer structure, as noted below.

All compounds exhibited high inhibitory activity against both AChE and BChE with selectivity toward the latter enzyme. We also observed mixed-type reversible inhibition of both cholinesterases. All compounds very weakly inhibited CES, which suggests the probable absence of undesirable drug–drug interactions arising from this potential source of hydrolytic biotransformation.

Compounds **3** with bis-*N*-acyl-alkylene spacers were more active inhibitors of both cholinesterases compared to compounds **5** with bis-thiourea-alkylene spacers. While the most active compounds **5** (**5c**–**e**) had anti-AChE and anti-BChE activity comparable or equal to amiridine, the most active compounds **3** (**3a**–**d**) exceeded both anti-AChE and anti-BChE activity of the parent compound. Compound **3c** was found to have a very high inhibitory activity against BChE, comparable to tacrine. Thus, for the first time, it was possible to obtain amiridine derivatives with inhibitory potencies against AChE and BChE that equaled or exceeded that of the parent compound, amiridine.

The lead compounds **3a**–**c**, with *N*-acyl-alkylene spacers of length *n* = 2, 3, 4, more effectively displaced propidium from the AChE PAS than the parent compound amiridine and the lead compounds **5c**–**e** with bis-thiourea-alkylene spacers.

Molecular docking explained the observed structure-inhibitory activity relationships. It also indicated binding of the conjugates to both principal sites in AChE, including the possibility of binding to the PAS, where the better binding affinity of compounds **3** (about 2 kcal/mol difference from compounds **5**) may result in the improved displacement of propidium iodide from the PAS by these compounds. These results, along with those from kinetics and propidium iodide displacement experiments, indicate that the conjugates **3** and **5** are dual-site binding AChE inhibitors that have the potential to block the AChE-induced aggregation of β-amyloid, which would be an ameliorating, disease-modifying effect.

Whereas the conjugates **3** containing bis-N-acyl-alkylene spacers have no effect on Aβ_42_ self-aggregation, this activity of the conjugates **5** depends on the length of the alkylene spacer. Only the compounds **5c**–**e** (*m* = 4, 5, 6) showed a pronounced inhibition of Aβ_42_ self-aggregation. Although the effect was rather weak compared to the reference compounds Myricetin and propidium iodide, it exceeded the anti-aggregation activity of the parent compound amiridine.

The most substantial difference between conjugates **3** and **5** is their antioxidant activity. Bis-amiridines **3** with N-acylalkylene spacers were almost inactive in the ABTS and FRAP tests, while compounds **5** with thiourea in the spacer demonstrated high antioxidant activity, especially in the ABTS test (TEAC = 1.2–2.1). This result is in agreement with the lower HOMO-LUMO gap values calculated for compounds **5**.

Our calculated ADMET profiles predicted good blood–brain barrier permeability, good intestinal absorption, and low cardiac toxicity risk for all compounds. Moreover, our predicted ADMET, physicochemical, and PAINS properties were acceptable for potential lead compounds at this phase of drug discovery.

Thus, the proposed approaches to amiridine molecule functionalization allowed us to obtain potent inhibitors of AChE and BChE with different pharmacological profiles, depending on spacer type, as potential multifunctional agents for the treatment of AD. Conjugates **3** with bis-N-acyl-alkylene spacers were more potent inhibitors of cholinesterases and AChE-induced aggregation of β-amyloid. The use of a thiourea-containing spacer results in bis-amiridines **5** with an expanded and more balanced pharmacological profile. Compounds **5c**–**5e** have emerged as the most interesting, as they displayed the properties of multitarget drug candidates for AD therapy. Not only do they have the potential to alleviate symptoms of AD, also showed an ability to act as antioxidants and to exert other potentially disease-modifying effects. Thus, we believe that these agents are worthy of further optimization and development as AD therapeutics.

## Figures and Tables

**Table 1:** Esterase profiles of conjugates **3, 5,** their ability to displace propidium from the *Ee*AChE PAS, and their inhibition of Aβ_42_ self-aggregation.

Compound		Inhibitory Activity Against AChE, BChE, and CES			Displacement of Propidium from *Ee*AChE PAS, (%) ^1^	Inhibition of Aβ_42_ Self-Aggregation, (%) ^2^
N	n/m	Human Erythrocyte AChE, IC_50_ (µM)	Equine Serum BChE, IC_50_ (µM)	Porcine Liver CES, (%) ^1^		
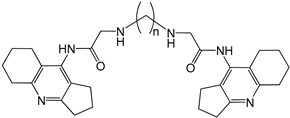						
**3a**	2	1.64 ± 0.08	0.110 ± 0.004	4.8 ± 0.4	14.4 ± 1.2	n.a.
**3b**	3	2.91 ± 0.74	0.128 ± 0.002	4.4 ± 0.5	14.7 ± 1.2	n.a.
**3c**	4	1.46 ± 0.03	0.067 ± 0.001	0.8 ± 0.2	17.7 ± 1.4	n.a.
**3d**	6	1.39 ± 0.10	1.11 ± 0.04	6.0 ± 0.6	11.2 ± 0.8	n.a.
**3e**	8	11.7 ± 1.1	6.65 ± 0.50	5.1 ± 0.9	13.2 ± 1.0	n.a.
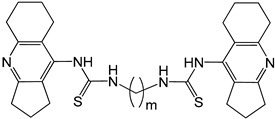						
**5a**	2	96.0 ± 3.7	4.08 ± 0.23	4.3 ± 0.2	7.6 ± 0.6	2.9 ± 0.2
**5b**	3	10.9 ± 1.0	0.689 ± 0.025	4.2 ± 0.5	9.3 ± 0.8	1.6 ± 0.1
**5c**	4	7.53 ± 0.59	0.802 ± 0.081	9.6 ± 0.8	10.0 ± 0.8	17.2 ± 1.5
**5d**	5	4.01 ± 0.13	0.758 ± 0.060	12.8 ± 1.1	11.2 ± 1.0	12.8 ± 1.1
**5e**	6	4.08 ± 0.46	0.769 ± 0.020	10.3 ± 1.0	11.1 ± 0.9	14.3 ± 1.1
**5f**	7	6.28 ± 0.46	1.18 ± 0.08	5.8 ± 0.3	2.5 ± 0.2	3.7 ± 0.3
**5g**	8	26.6 ± 0.6	1.45 ± 0.09	15.9 ± 1.5	2.2 ± 0.2	5.2 ± 0.4
**Reference Agents**						
**Amiridine**		4.44 ± 0.36	0.272 ± 0.015	2.7 ± 0.5	12.2 ± 0.9	6.4 ± 0.5
**Tacrine**		0.601 ± 0.047	0.0295 ± 0.002	n.a.	3.1 ± 0.2	5.9 ± 0.5
**BNPP**		n.a.	n.a.	99.1 ± 0.93 ^3^	n.d.	n.d.
**Donepezil**		0.040 ± 0.004	19.2 ± 3.0	n.a.	11.9 ± 0.9	n.d.
**Myricetin**		n.d.	n.d.	n.d.	n.d.	74.7 ± 5.2
**Propidium iodide**		n.d.	n.d.	n.d.	n.d.	90.7 ± 7.1

^1^ compound concentration 20 µM. ^2^ Inhibition of Aβ_42_ self-aggregation (50 µM) by the tested compound at 100 µM concentration. ^3^ BNPP IC_50_ CES = 1.80 ± 0.11 µM. n.d.—not determined. n.a.—not active. Data are expressed as mean ± SEM, *n* = 3.

**Table 2:** Antioxidant properties of conjugates **3**, **5**.

Compound		ABTS^•+^-Scavenging Activity		FRAP, TE ^2,3^	HOMO-LUMO Gap, eV
N	n/m	TEAC ^1^	IC_50_, µM		
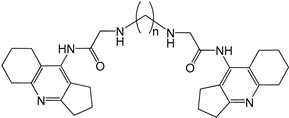					
**3a**	2	0.10 ± 0.004	~180	n.a.	5.32
**3b**	3	0.05 ± 0.003	>200	0.05 ± 0.01	5.32
**3c**	4	0.10 ± 0.005	~200	n.a.	5.36
**3d**	6	n.a.	n.d.	n.a.	5.16
**3e**	8	0.04 ± 0.002	>200	0.05 ± 0.01	5.42
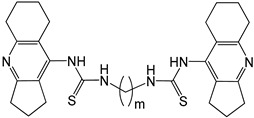					
**5a**	2	2.1 ± 0.07	7.6 ± 0.4	0.52 ± 0.04	4.50
**5b**	3	1.9 ± 0.08	9.9 ± 0.5	0.50 ± 0.03	4.44
**5c**	4	1.8 ± 0.05	9.7 ± 0.6	0.35 ± 0.02	4.42
**5d**	5	1.6 ± 0.06	10.9 ± 0.7	0.45 ± 0.03	4.39
**5e**	6	1.5 ± 0.07	10.9 ± 0.7	0.49 ± 0.01	4.45
**5f**	7	1.2 ± 0.05	12.4 ± 0.8	0.55 ± 0.01	4.38
**5g**	8	1.5 ± 0.07	10.8 ± 0.6	0.85 ± 0.03	4.40
**Reference Agents**					
**Amiridine**		0.04 ± 0.003	n.d.	n.a.	n.d.
**Trolox**		1.0	20.1 ± 1.2	1.0	n.d.

^1^ Trolox equivalent antioxidant capacity (TEAC) was determined from the ratio of the slopes of the concentration–response curves [(test compound)/Trolox]. ^2^ TE—Trolox equivalents, the values calculated as the ratio of the concentrations of Trolox and the test compound resulting in the same effect in the FRAP test. ^3^ Compound concentration 20 µM. Data are expressed as mean ± SEM, *n* = 3.

**Table 3:** Predicted ADMET and physicochemical profiles of compounds **3** and **5**.

Compound		MW	LogP_ow_	pS	LogBB	HIA, %	hERG, *pK_i_*	hERG, *pIC_50_*	QED
N	*n/m*								
**3a**	2	516.69	1.56	3.36	−1.31	66	4.15	4.39	0.38
**3b**	3	530.72	1.59	3.57	−1.45	66	4.28	4.59	0.35
**3c**	4	544.74	1.91	3.86	−1.39	66	4.37	4.66	0.32
**3d**	6	572.80	2.53	4.08	−1.32	66	4.19	4.92	0.26
**3e**	8	600.85	3.26	4.98	−1.02	66	4.55	5.28	0.21
**5a**	2	520.76	3.82	5.34	−1.36	57	4.46	4.78	0.34
**5b**	3	534.79	4.14	5.57	−1.33	57	4.65	5.19	0.31
**5c**	4	548.81	4.51	6.01	−1.31	57	4.69	5.10	0.28
**5d**	5	562.84	4.91	6.29	−1.28	57	4.73	5.26	0.24
**5e**	6	576.87	5.30	6.55	−1.37	57	4.50	5.38	0.21
**5f**	7	590.89	5.70	6.81	−1.16	57	4.82	5.60	0.19
**5g**	8	604.92	6.07	6.97	−1.07	57	4.86	5.78	0.16
**Amiridine**		188.27	2.62	1.75	−0.58	92	4.34	4.44	0.68
**Tacrine**		198.27	2.95	1.52	−0.00	93	4.98	4.98	0.71

MW—molecular weight, LogP_ow_—octanol-water partition coefficient, pS—aqueous solubility [−log(M)], LogBB—blood–brain barrier distribution, HIA—human intestinal absorption [%], hERG *pKi*—hERG potassium channel affinity [−log(M)], hERG, *pIC_50_*—hERG potassium channel inhibitory activity [−log(M)], QED—quantitative estimate of drug-likeness.

## Data Availability

Not applicable.

## Supplementary Materials

The following are available online: (1) Figures S1–S23—NMR spectra for **3a**–**e**, **5a**–**g**; (2) Figures S24 and S25—original graphs for IC_50_ assay, (3) Figures S26 and S27—supplementary molecular docking studies; (4) Tables S1 and S2—supplementary information on p*K*_a_ estimations.

## Sample Availability

Samples of the compounds are not available.
