# Supplementary material for: Bis-Amiridines as Acetylcholinesterase and Butyrylcholinesterase Inhibitors: N-Functionalization Determines the Multitarget Anti-Alzheimer’s Activity Profile
Source: Molecules. 2022 Feb 4;27(3):1060. doi: 10.3390/molecules27031060 (PMC8839189; doi:10.3390/molecules27031060)
Supplement: Supplementary file 1 [file molecules-27-01060-s001.zip › FigS24-S25_Suppl_IC50.pdf]

Figure S24. IC_{50} values for AChE inhibition by compounds **3,5** (MEAN ± SEM, n = 3)

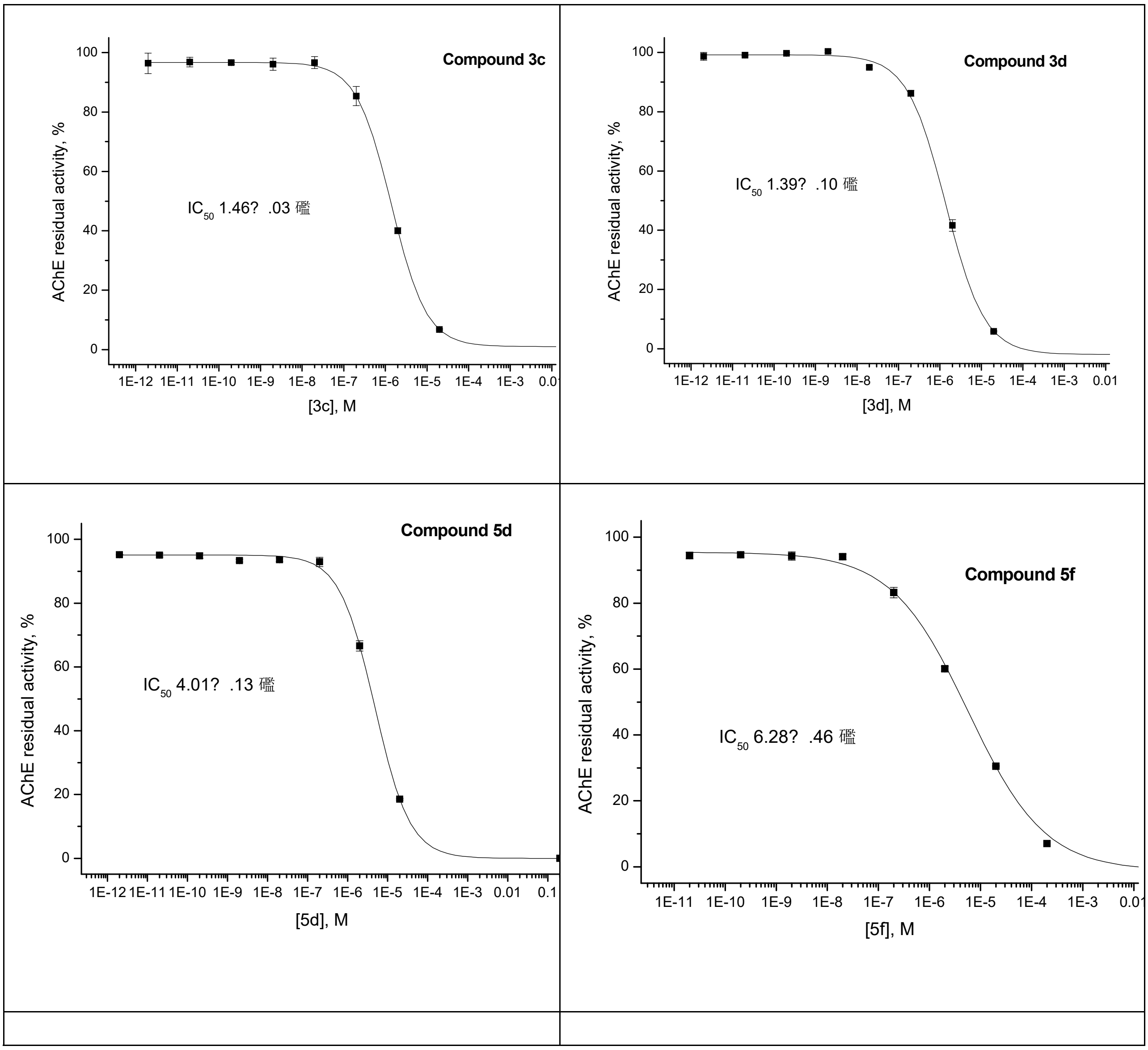

Figure S25. IC_{50} values for BChE inhibition by compounds **3,5** (MEAN ± SEM, n = 3)

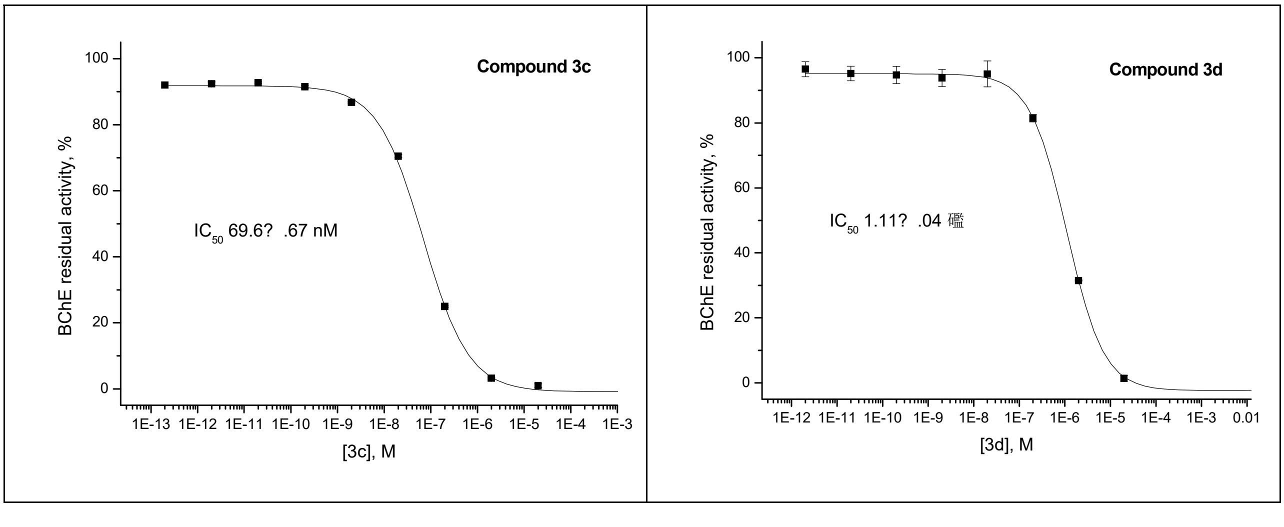

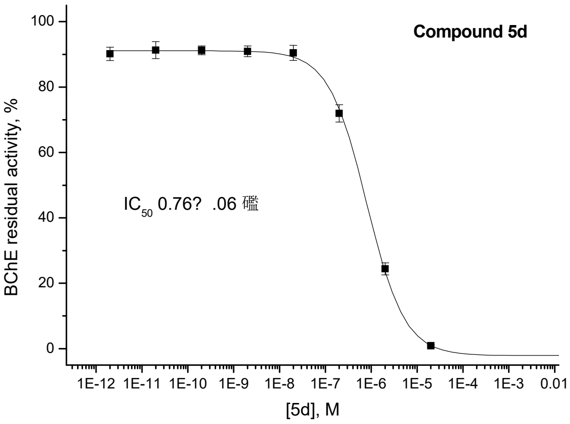
Compound 5d
IC50 0.76? .06 礛
BChE residual activity, %
100
80
60
40
20
0
1E-12 1E-11 1E-10 1E-9 1E-8 1E-7 1E-6 1E-5 1E-4 1E-3 0.01
[5d], M

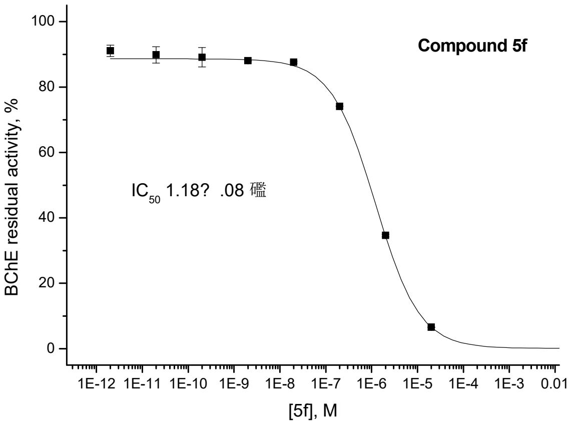
Compound 5f
IC50 1.18? .08 礛
BChE residual activity, %
100
80
60
40
20
0
1E-12 1E-11 1E-10 1E-9 1E-8 1E-7 1E-6 1E-5 1E-4 1E-3 0.01
[5f], M
