# Supplementary material for: Bis-Amiridines as Acetylcholinesterase and Butyrylcholinesterase Inhibitors: N-Functionalization Determines the Multitarget Anti-Alzheimer’s Activity Profile
Source: Molecules. 2022 Feb 4;27(3):1060. doi: 10.3390/molecules27031060 (PMC8839189; doi:10.3390/molecules27031060)
Supplement: Supplementary file 1 [file molecules-27-01060-s001.zip › FigS26_FIGS27_Docking.pdf]

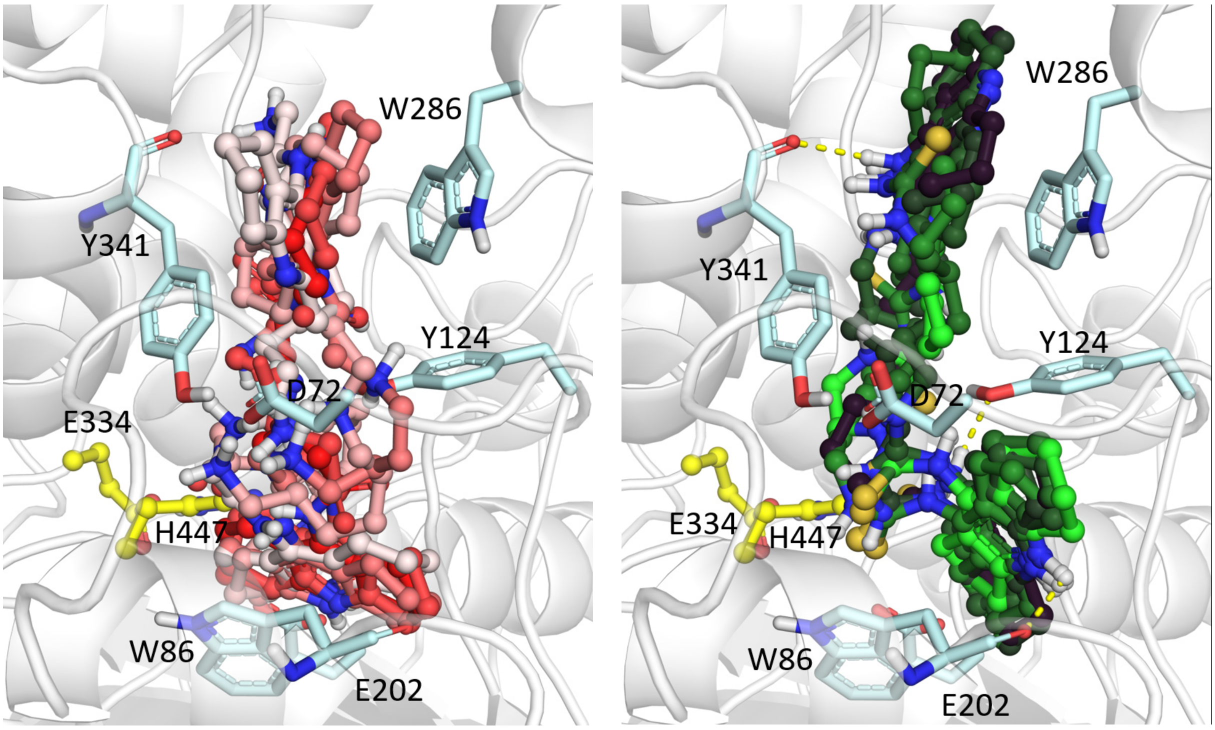


(a) (b)

**Figure S26.** Molecular docking of compounds **3** (A, carbon atoms are shown in shades of red) and **5** (B, carbon atoms are shown in shades of green) into AChE active site.

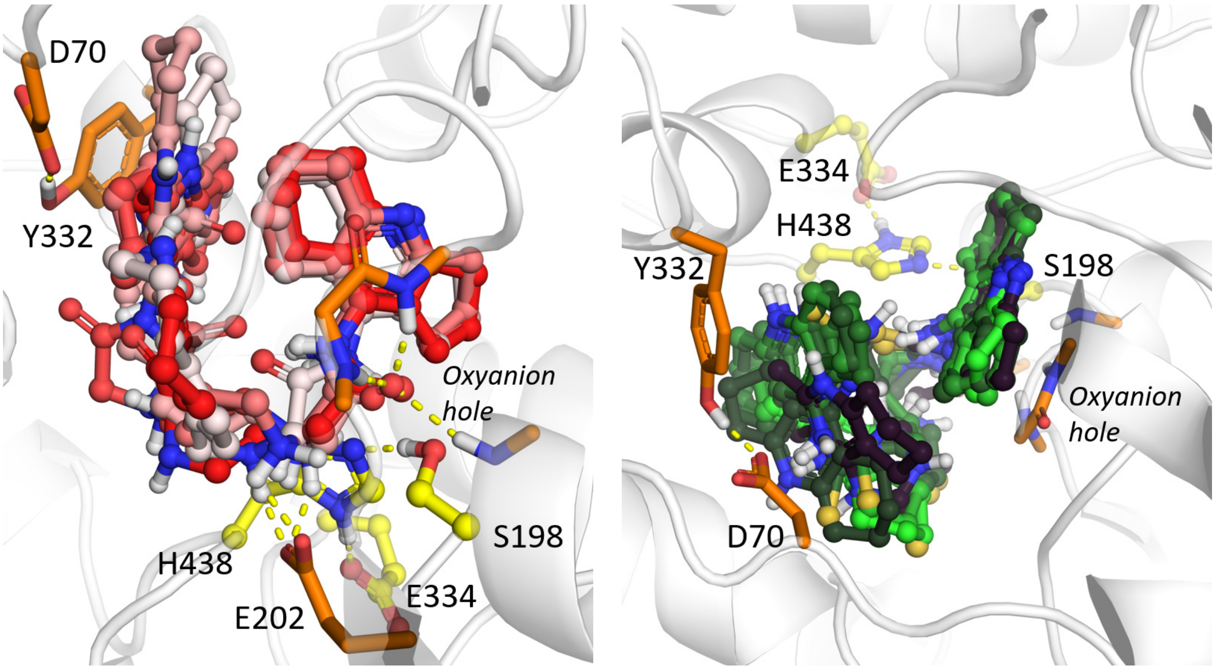


(a) (b)

**Figure S27.** Molecular docking of compounds **3** (A, carbon atoms are shown in shades of red) and **5** (B, carbon atoms are shown in shades of green) into BChE active site.
