# Supplementary material for: Bis-Amiridines as Acetylcholinesterase and Butyrylcholinesterase Inhibitors: N-Functionalization Determines the Multitarget Anti-Alzheimer’s Activity Profile
Source: Molecules. 2022 Feb 4;27(3):1060. doi: 10.3390/molecules27031060 (PMC8839189; doi:10.3390/molecules27031060)
Supplement: Supplementary file 1 [file molecules-27-01060-s001.zip › Suppl_Makhaeva_Table_of_Contents.pdf]

Bis-Amiridines as Acetylcholinesterase and Butyrylcholines-terase Inhibitors: N-Functionalization Determines the Multi-target Anti-Alzheimer's Activity Profile

Galina F. Makhaeva [1], Nadezhda V. Kovaleva [1], Natalia P. Boltneva [1], Elena V. Rudakova [1], Sofya V. Lushchekina [1,2], Tatiana Yu. Astakhova [2], Igor V. Serkov [1], Alexey N. Proshin [1], Eugene V. Radchenko [3], Vladimir A. Palyulin [3], Jan Korabecny [4], Ondrej Soukup [4], Sergey O. Bachurin [1] and Rudy J. Richardson [5,6,7,8,*]

1 Institute of Physiologically Active Compounds, Russian Academy of Sciences, 142432 Chernogolovka, Russia; gmakh@ipac.ac.ru (G.F.M.); kovalevanv@ipac.ac.ru (N.V.K.); boltneva@ipac.ac.ru (N.P.B.); rudakova@ipac.ac.ru (E.V.R.); sofya.lushchekina@gmail.com (S.V.L.); serkoviv@mail.ru (I.V.S.); proshin@ipac.ac.ru (A.N.P.); bachurin@ipac.ac.ru (S.O.B.)
2 Emanuel Institute of Biochemical Physics, Russian Academy of Sciences, 119334 Moscow, Russia; astakhova1967.t@yandex.ru
3 Department of Chemistry, Lomonosov Moscow State University, 119991 Moscow, Russia; genie@qsar.chem.msu.ru (E.V.R.); vap@qsar.chem.msu.ru (V.A.P.)
4 Biomedical Research Centre, University Hospital Hradec Kralove, 500 05 Hradec Kralove, Czech Republic; jan.korabecny@fnhk.cz (J.K.), ondrej.soukup@fnhk.cz (O.S.)
5 Department of Environmental Health Sciences, University of Michigan, Ann Arbor, MI 48109, USA
6 Department of Neurology, University of Michigan, Ann Arbor, MI 48109, USA
7 Center of Computational Medicine and Bioinformatics, University of Michigan, Ann Arbor, MI 48109, USA
8 Michigan Institute for Computational Discovery and Engineering, University of Michigan, Ann Arbor, MI 48109, USA
* Correspondence: rjrich@umich.edu; Tel.: +1-734-936-0769

Supplementary Materials: The following are available online: 1) NMR spectra for **3a-e**, **5a-g**; 2) original graphs for IC_{50} assay, 3) Table S1: supplementary information on pK_a estimations, and 4) supplementary molecular docking studies.

Table of contents

**Figure S1:** NMR spectra for **3a**	
**Figure S2:** NMR spectra for **3b**	
**Figure S3:** NMR spectra for **3b_13C**	
**Figure S4:** NMR spectra for **3c**	
**Figure S5:** NMR spectra for **3d**	
**Figure S6:** NMR spectra for **3e**	
**Figure S7:** NMR spectra for **3e_13C**	
**Figure S8:** NMR spectra for **4**	
**Figure S9:** NMR spectra for **4_13C**	
**Figure S10:** NMR spectra for **5a**	
**Figure S11:** NMR spectra for **5a_13C**	
**Figure S12:** NMR spectra for **5b**	
**Figure S13:** NMR ^{13}C spectra for **5b_13C**	
**Figure S14:** NMR ^{13}C spectra for **5c**	

Figure S15: NMR ^{13}C spectra for **5c_13C**

Figure S16: NMR ^{13}C spectra for **5d**

Figure S17: ^{13}C NMR spectra for **5d_13C**

Figure S18: ^{13}C NMR spectra for **5e**

Figure S19: ^{13}C NMR spectra for **5e_13C**

Figure S20: ^{13}C NMR spectra for **5f**

Figure S21: ^{13}C NMR spectra for **5f_13C**

Figure S22: ^{13}C NMR spectra for **5g**

Figure S23: ^{13}C NMR spectra for **5g_13C**

Figure S24. IC_{50} values for hAChE inhibition by compounds **3** and **5,** (MEAN ± SEM, n = 3)

Figure S25. IC_{50} values for eqBChE inhibition by **3** and **5,** (MEAN ± SEM, n = 3)

Figure S26. Molecular docking of compounds 3 and **5** into AChE active site.

Figure S27. Molecular docking of compounds 3 and **5** into BChE active site.

Table S1-2. Estimated pK_a values of bis-amiridines **3** and **5**
