# Supplementary figures and images for: Bis-Amiridines as Acetylcholinesterase and Butyrylcholinesterase Inhibitors: N-Functionalization Determines the Multitarget Anti-Alzheimer’s Activity Profile
Source: Molecules. 2022 Feb 4;27(3):1060. doi: 10.3390/molecules27031060 (PMC8839189; doi:10.3390/molecules27031060)

**Figures S1-S23: NMR spectra for 3a-e, 5a-g**

**3a**

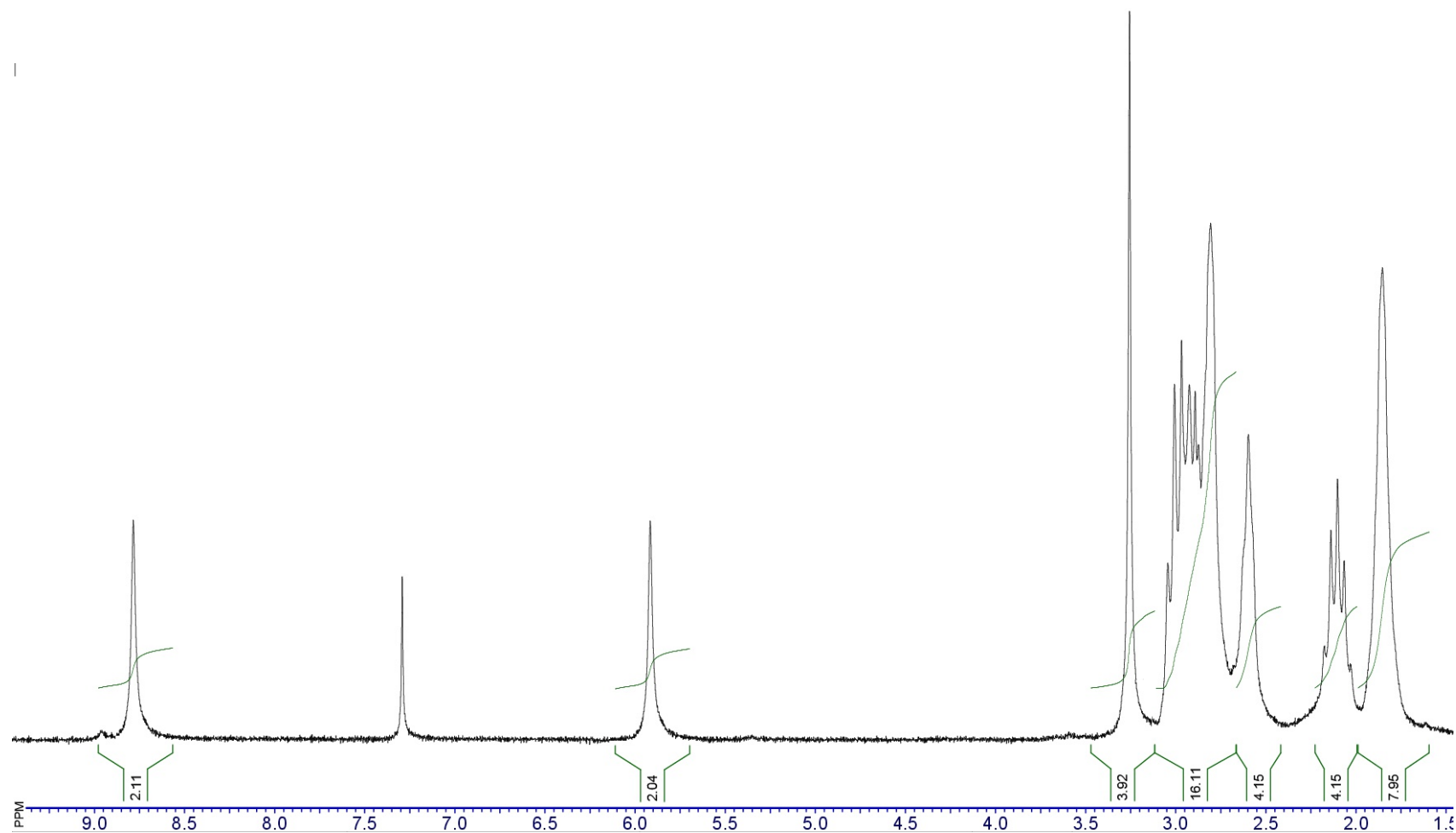

3b

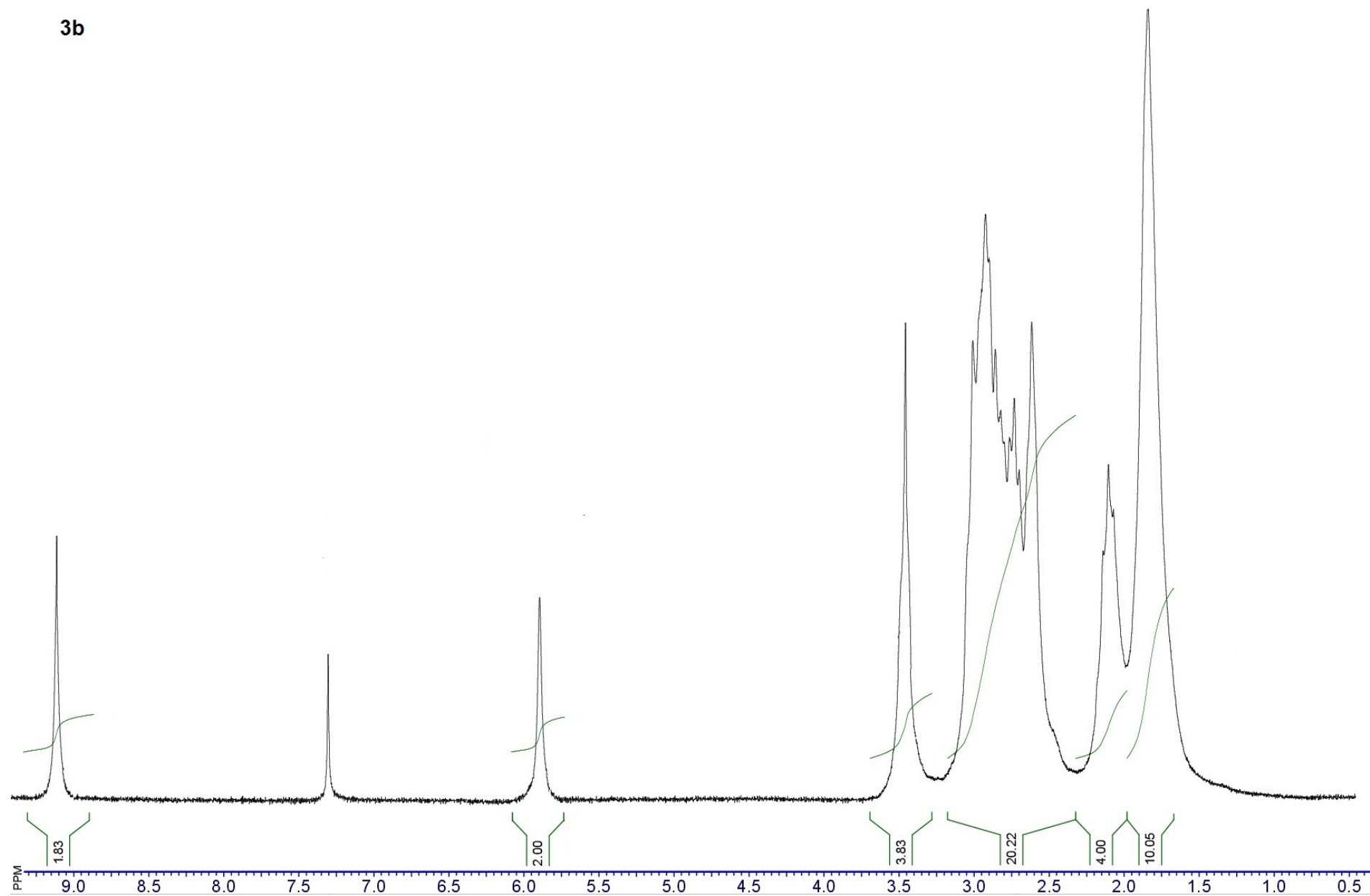

3b\_13C

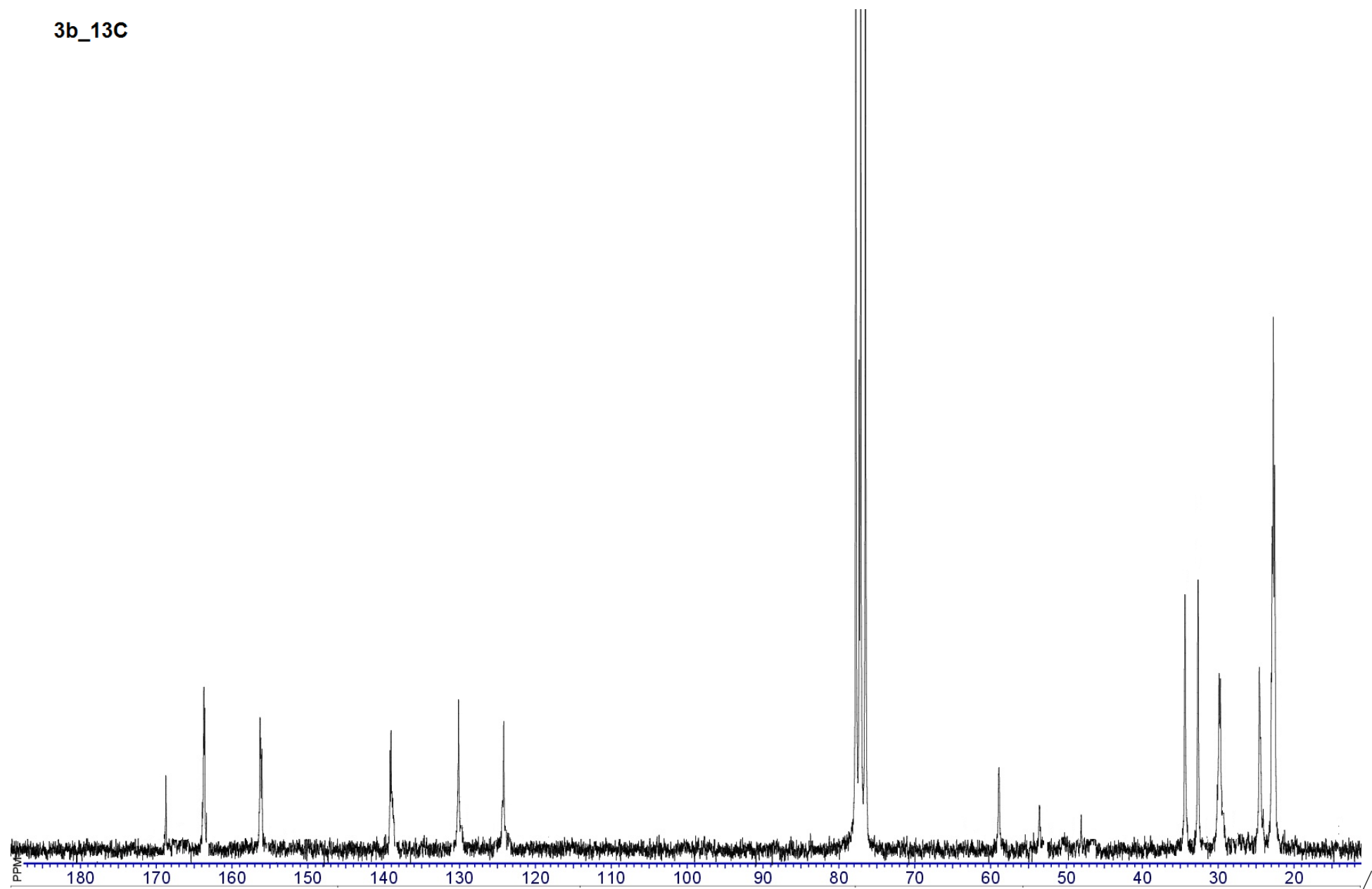

3d

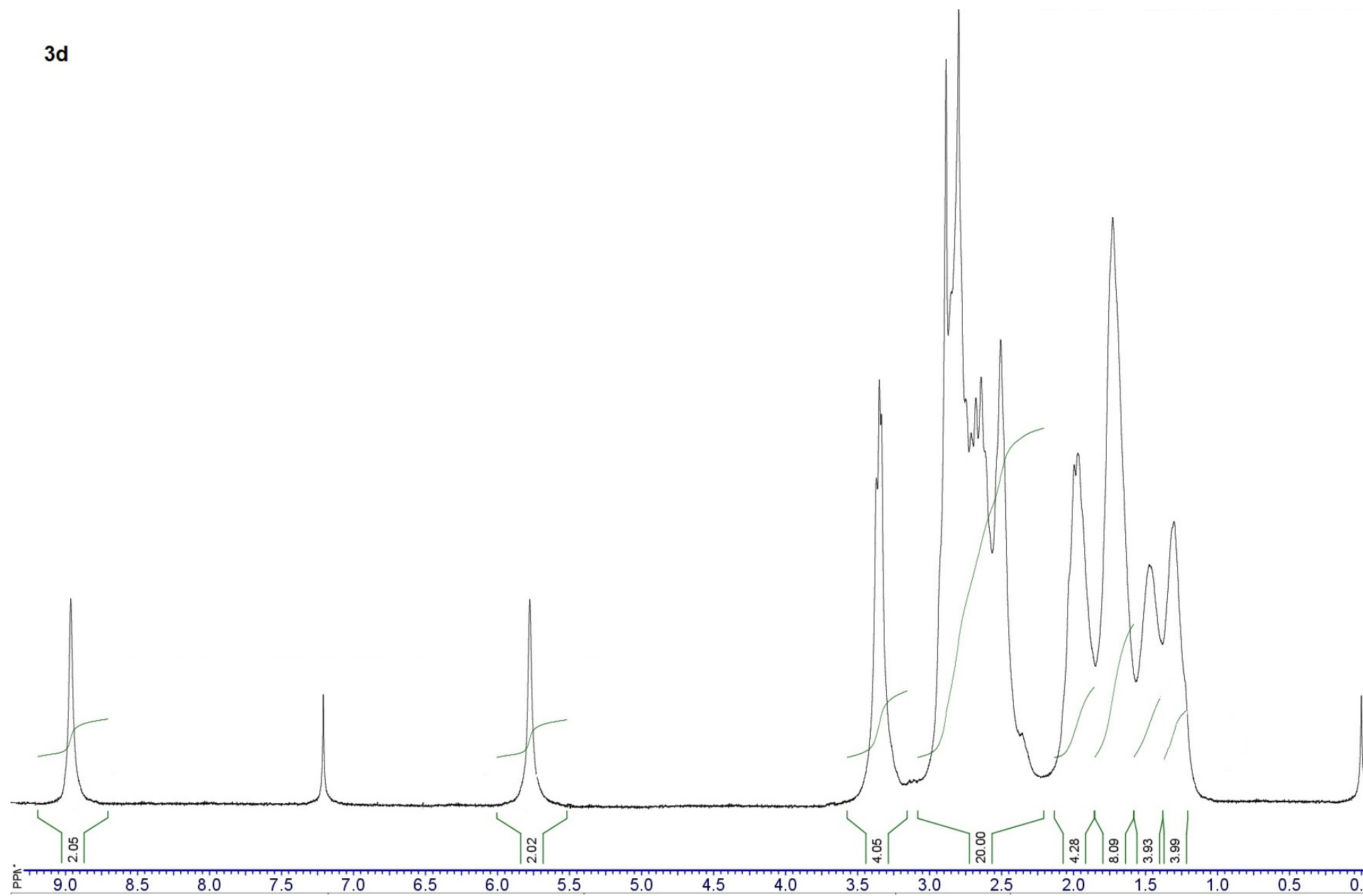

3e

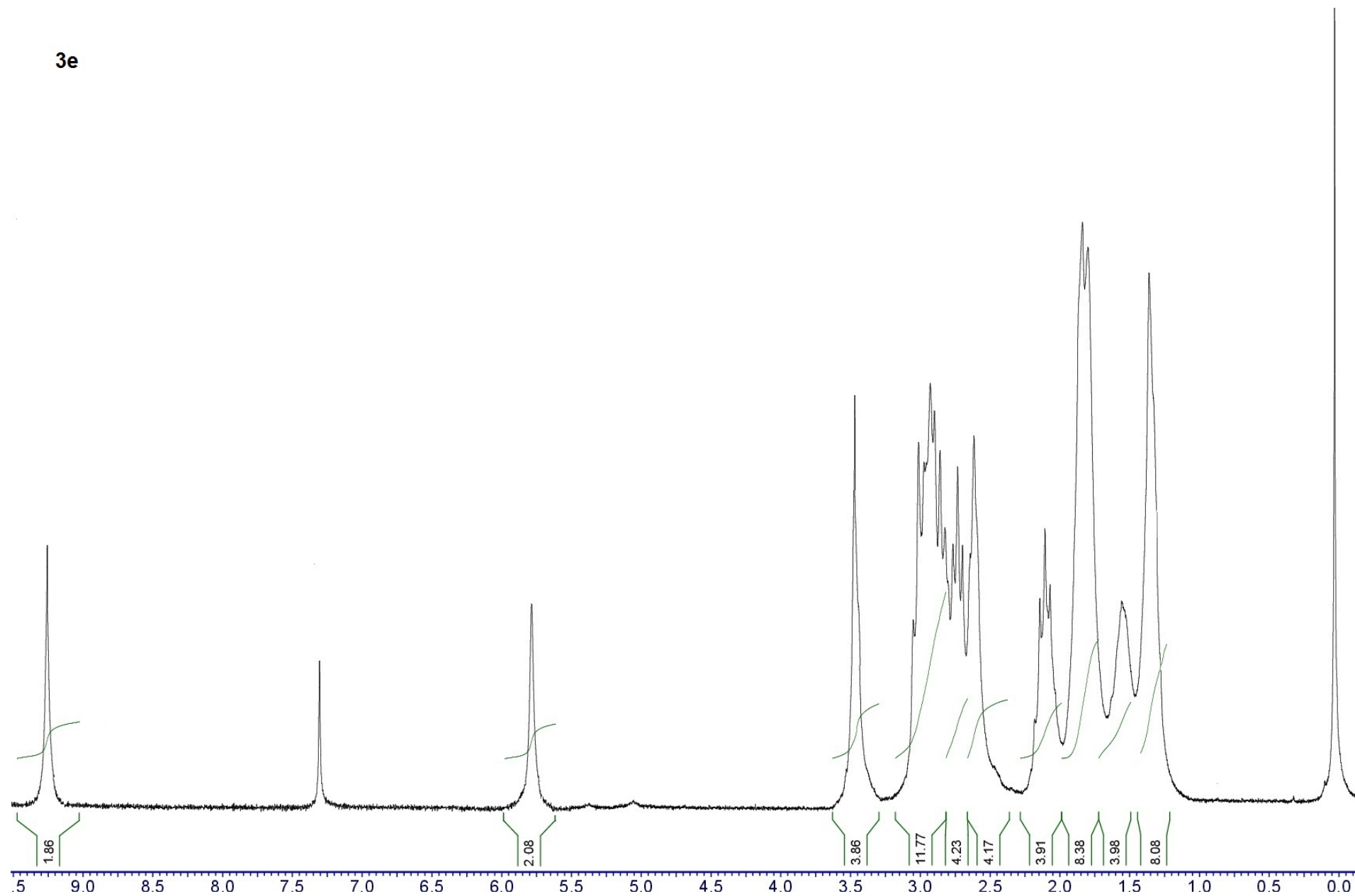

3e\_13C

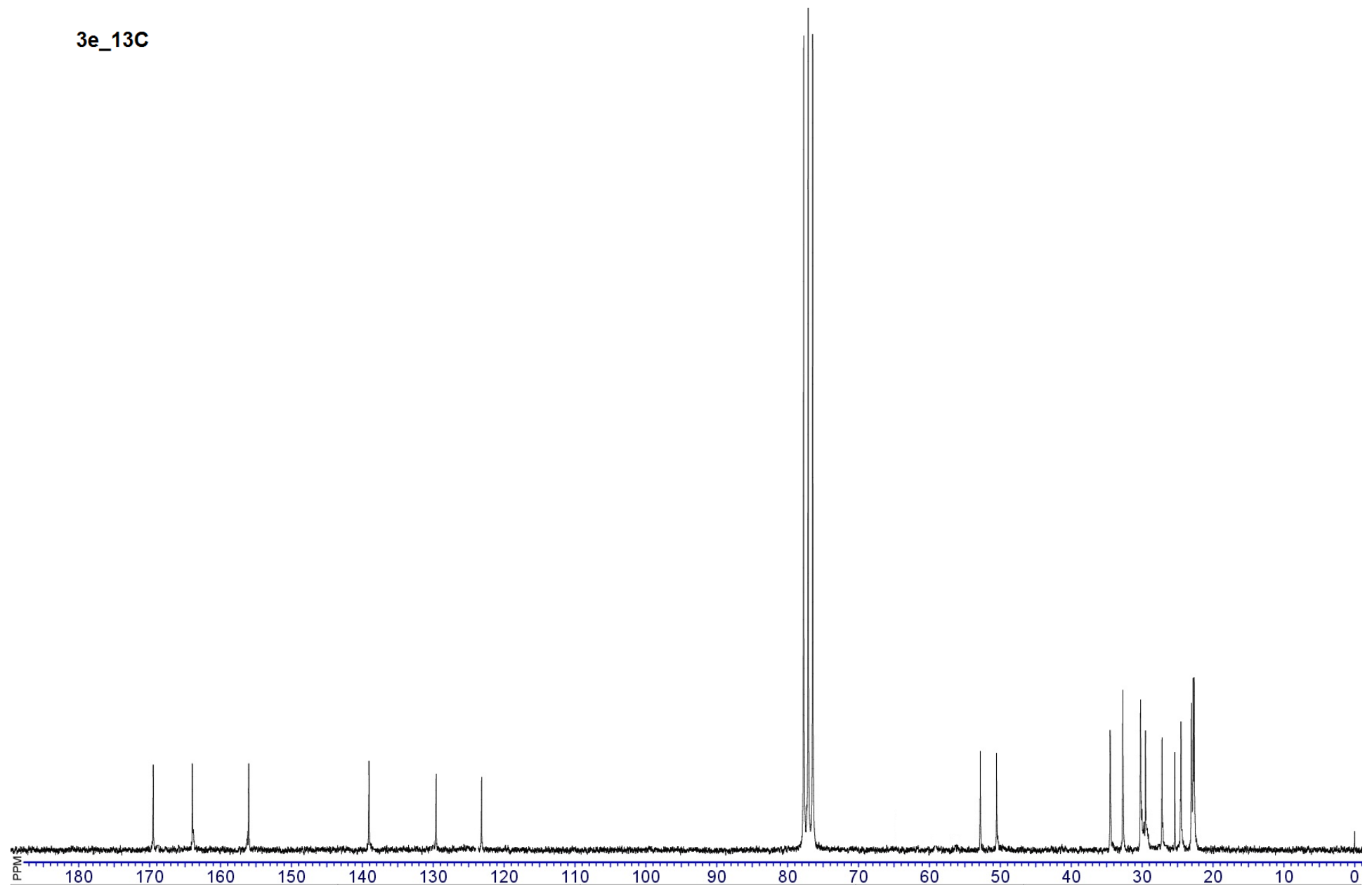

4

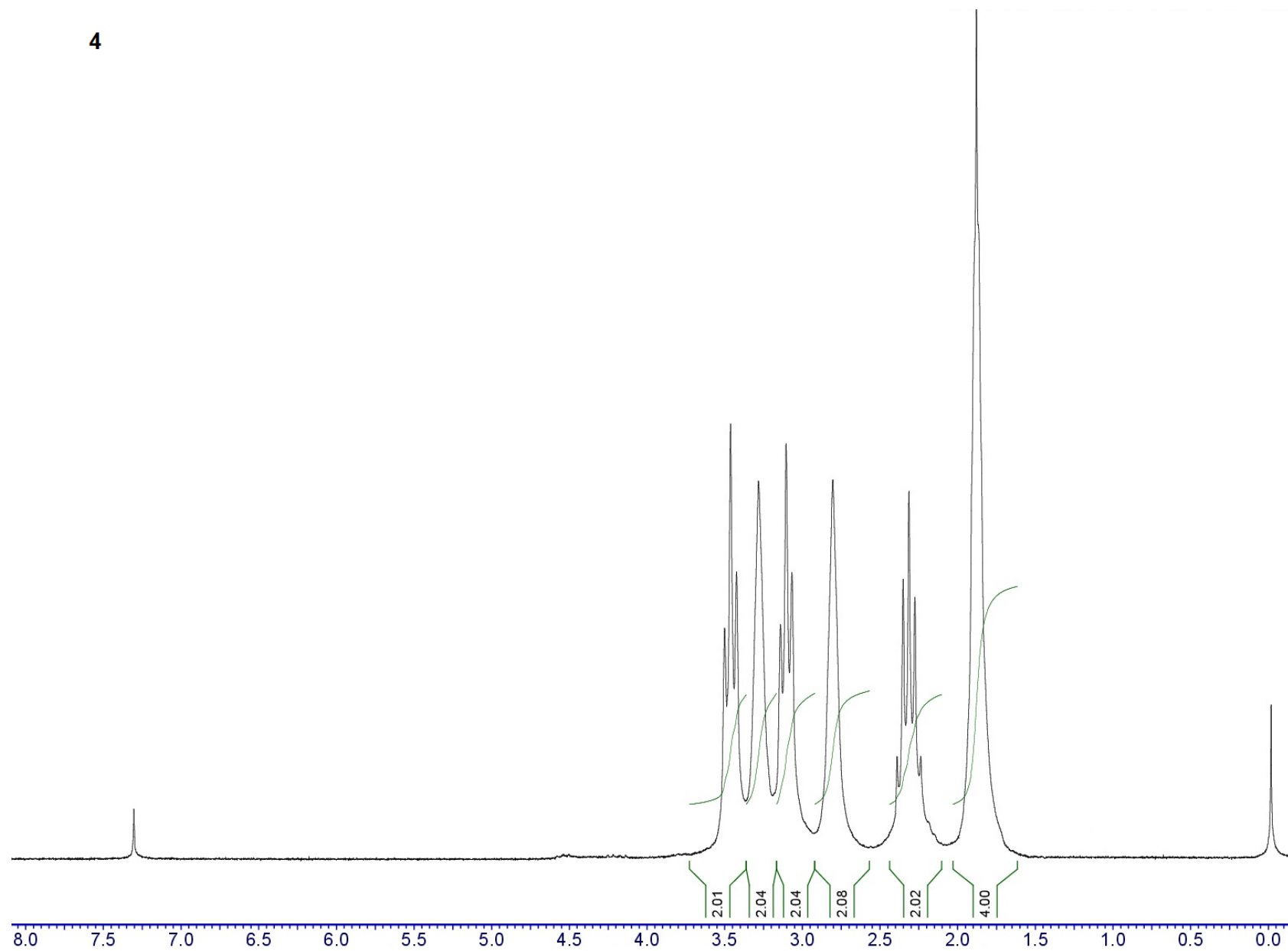

4\_13C

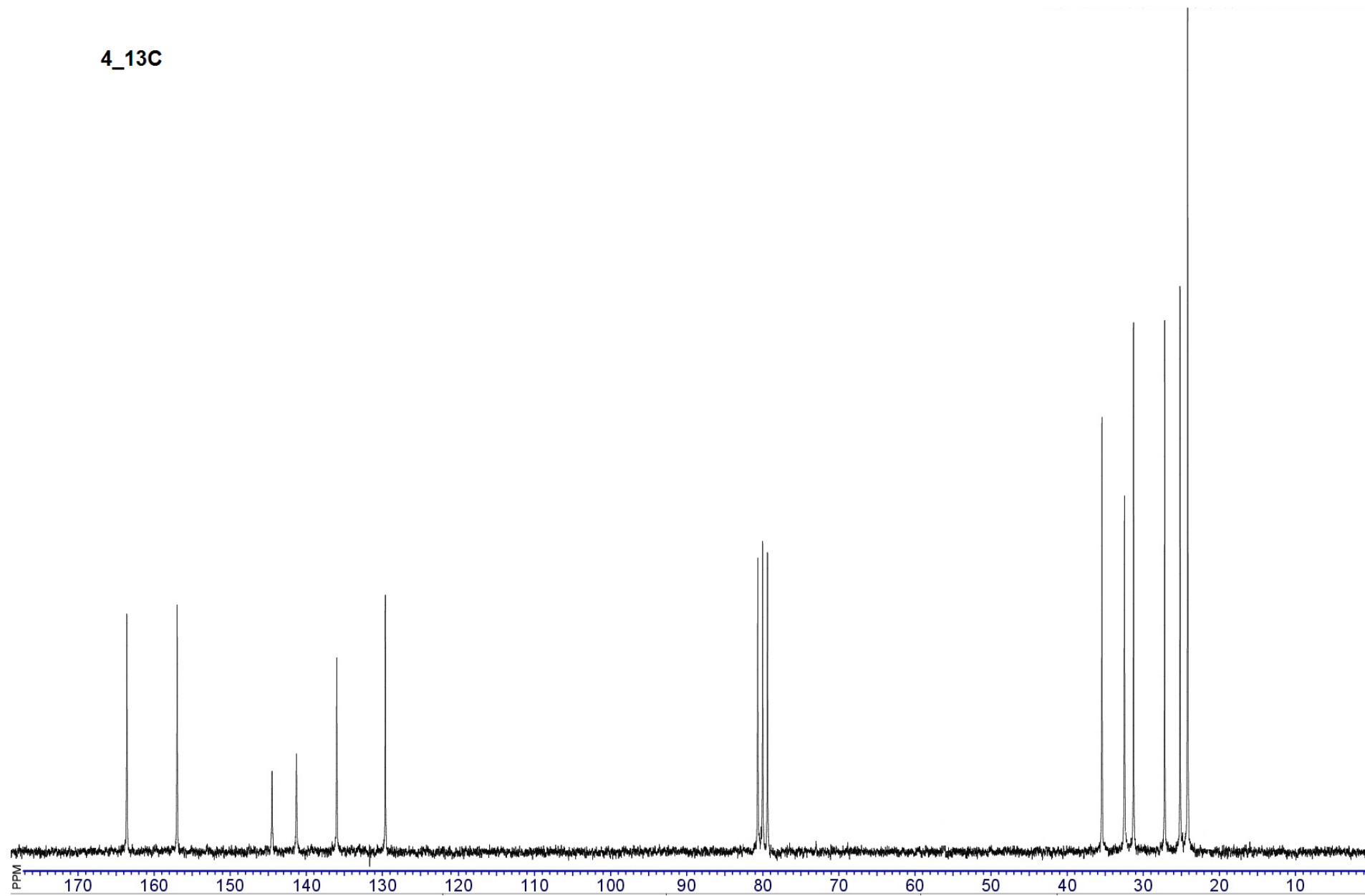

5a

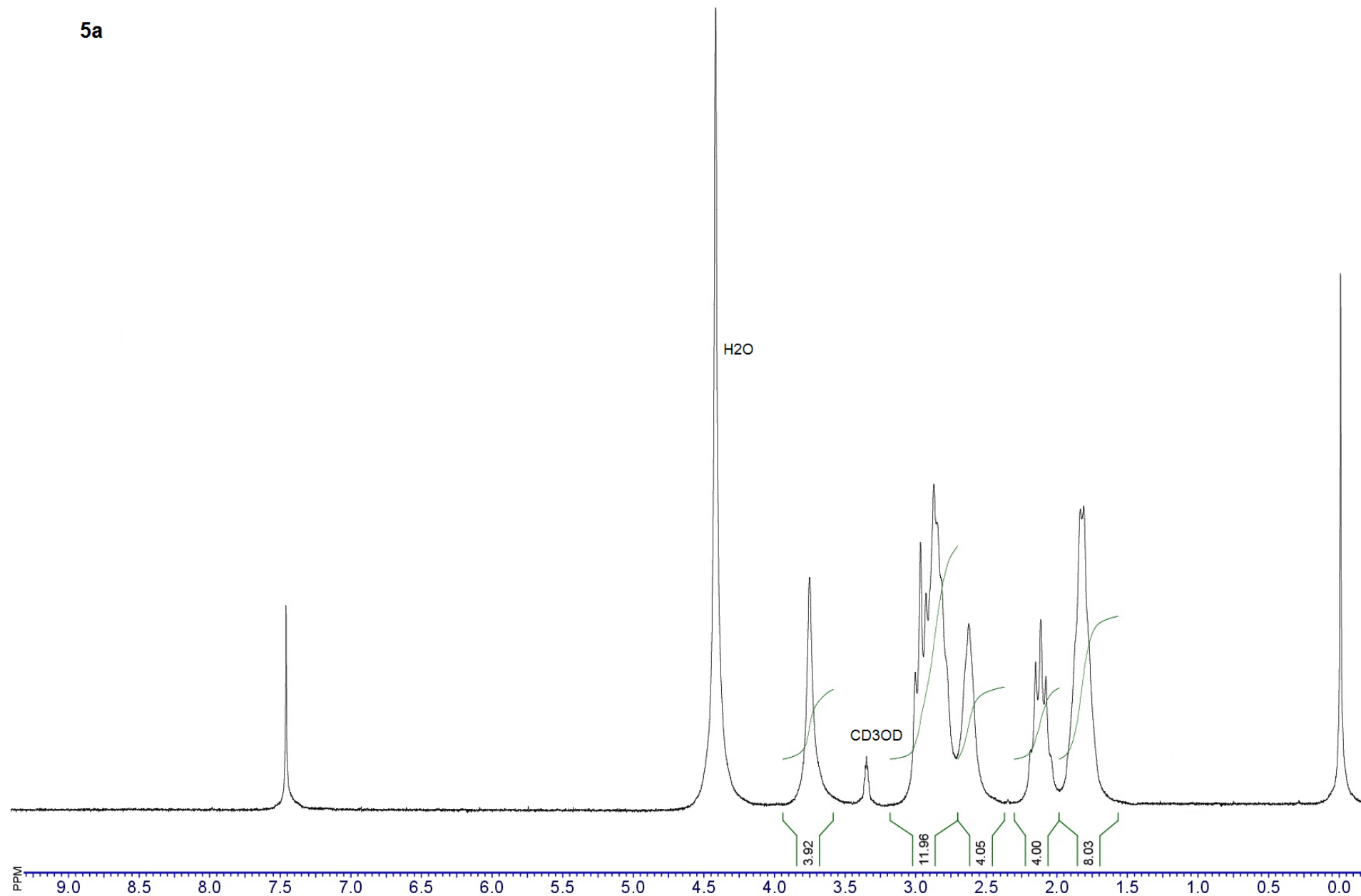

5a\_13C

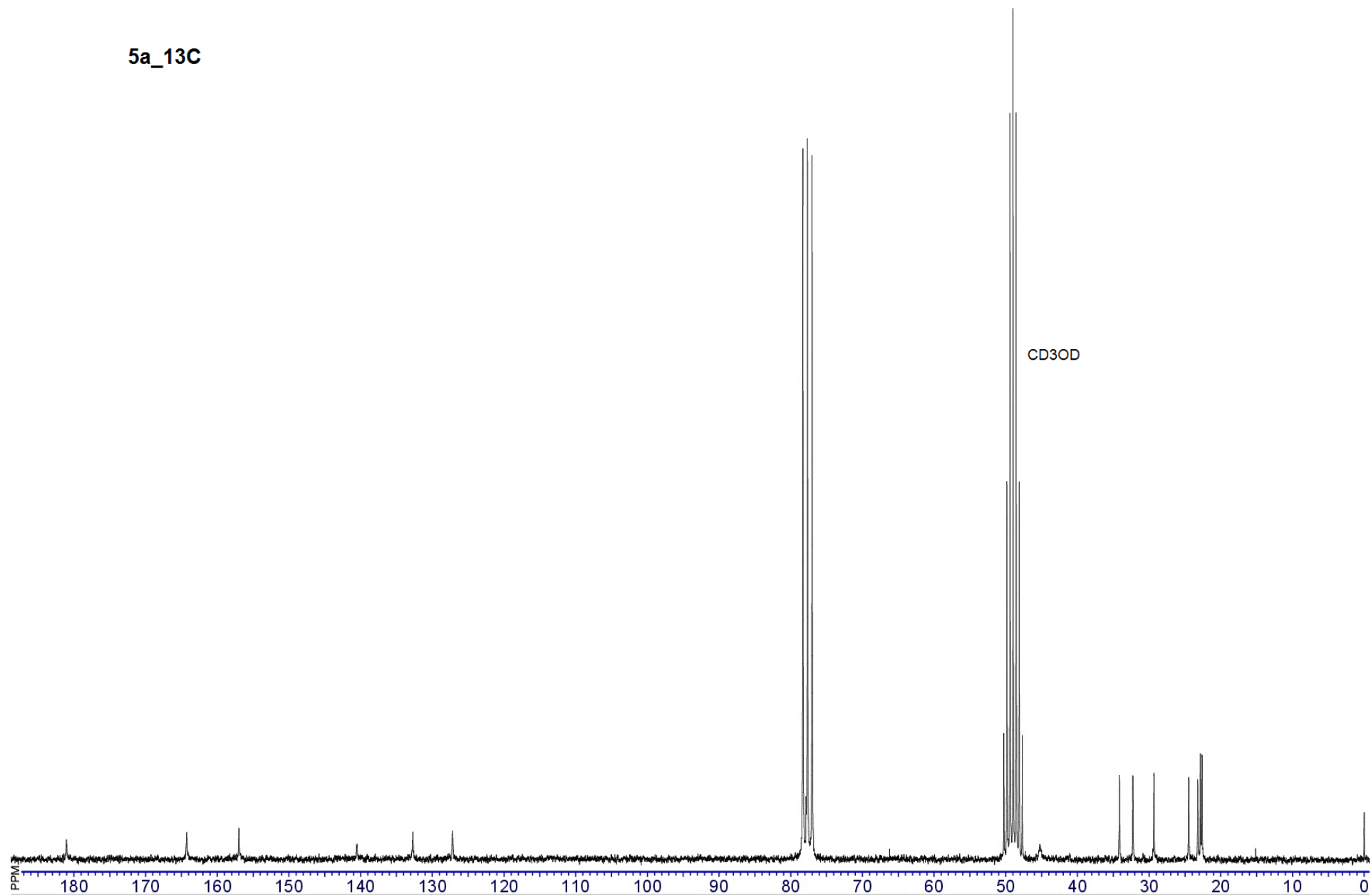

5b

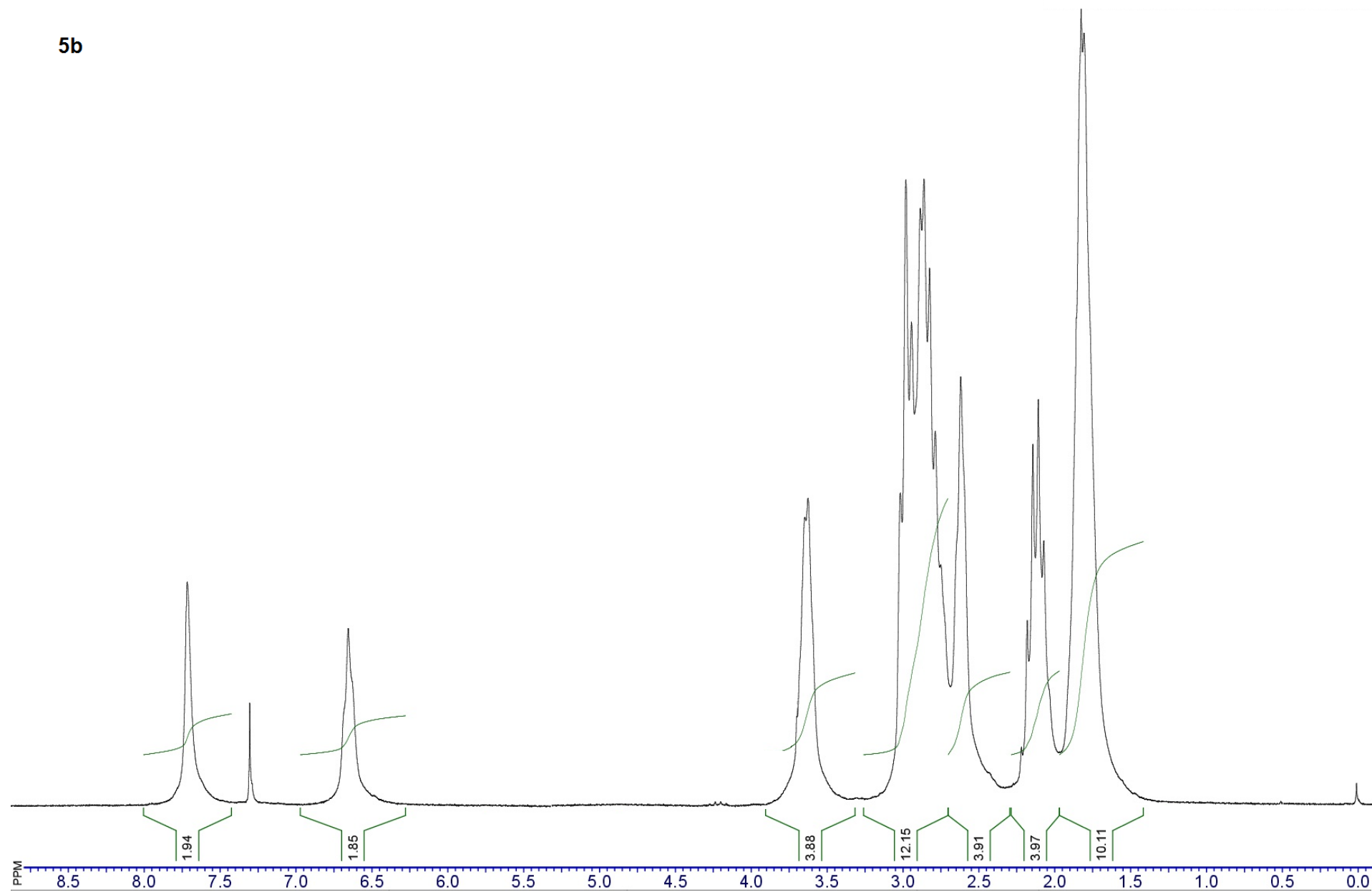

5b\_13C

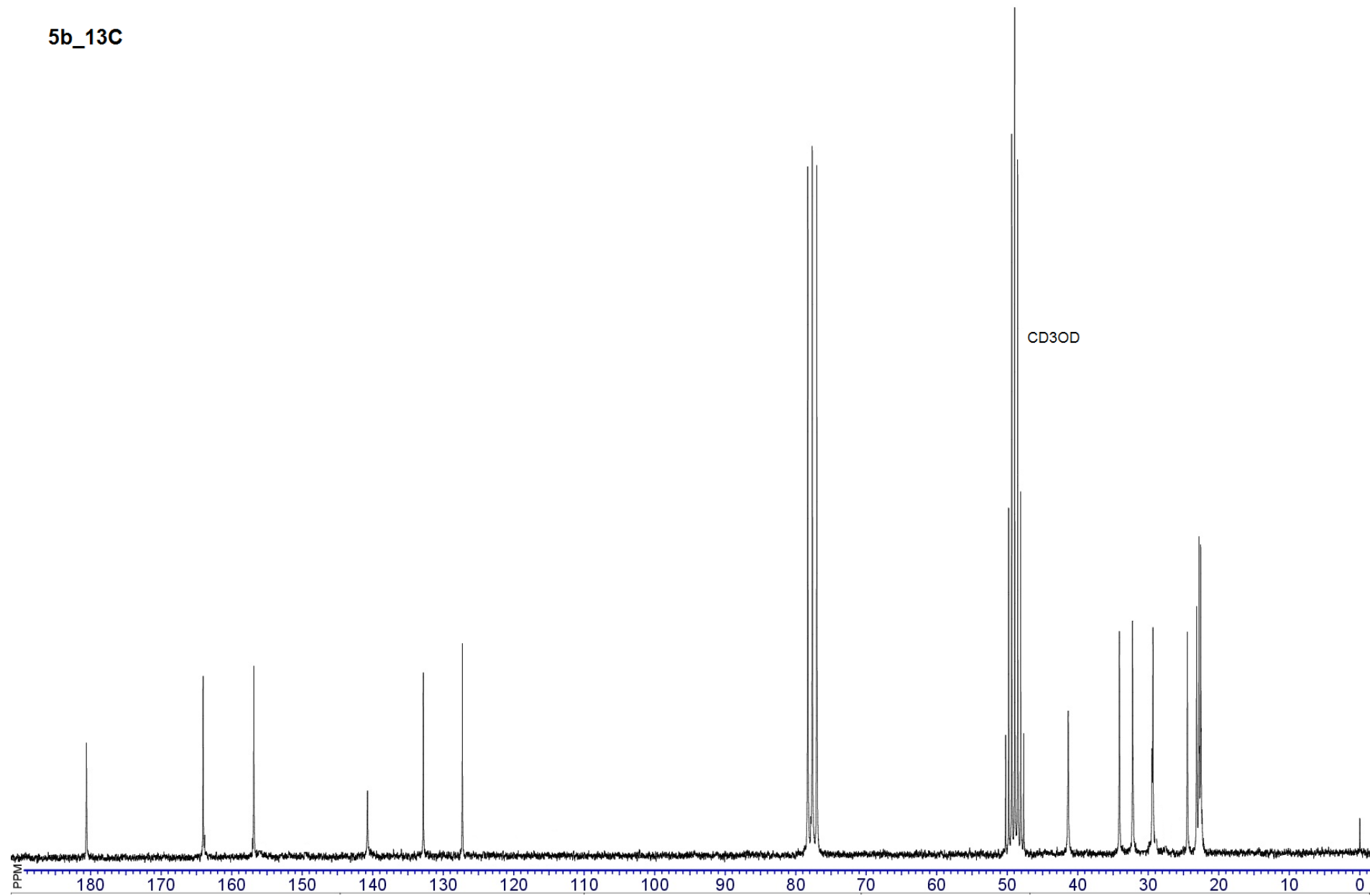

5c

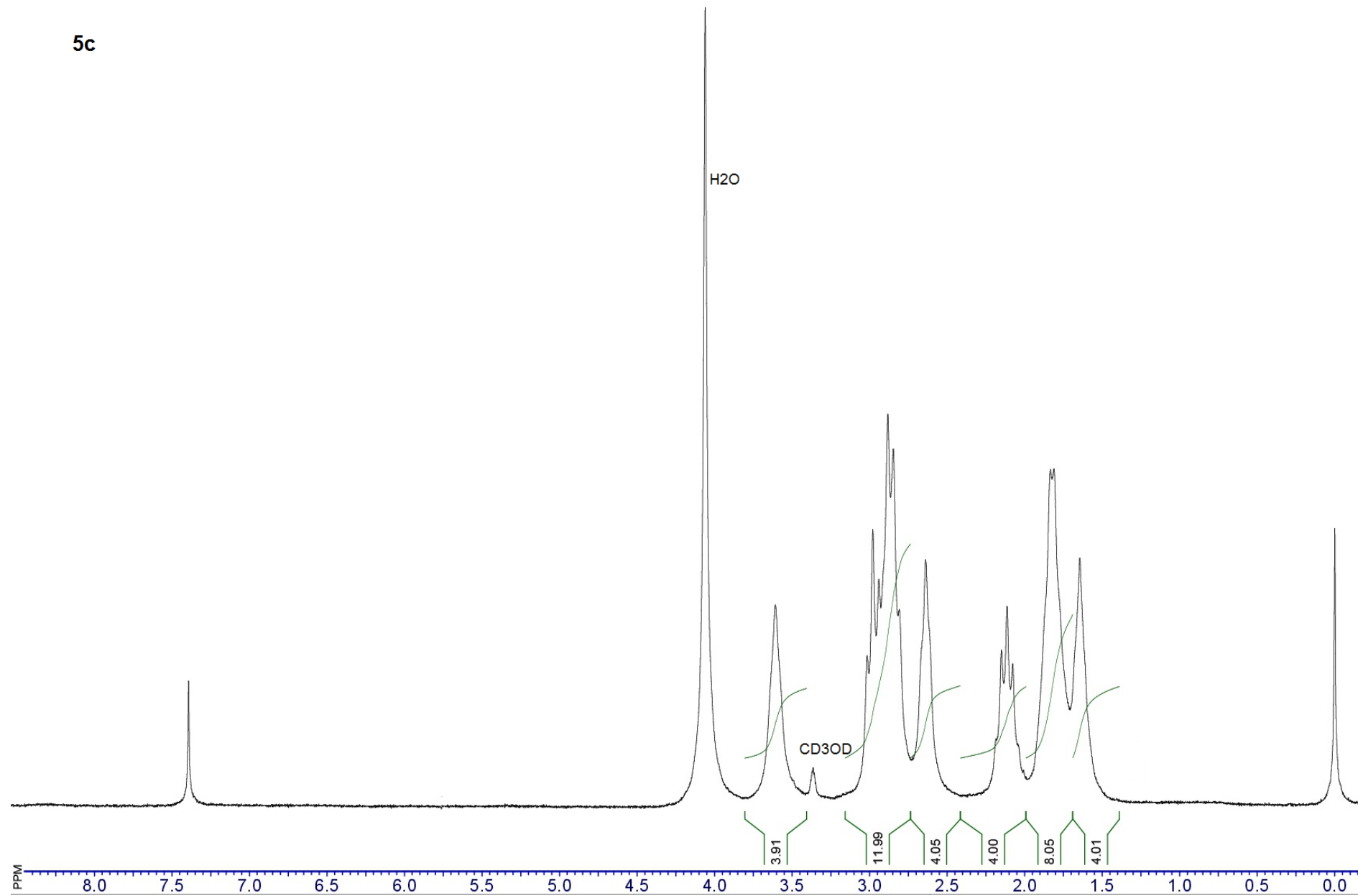

5c\_13C

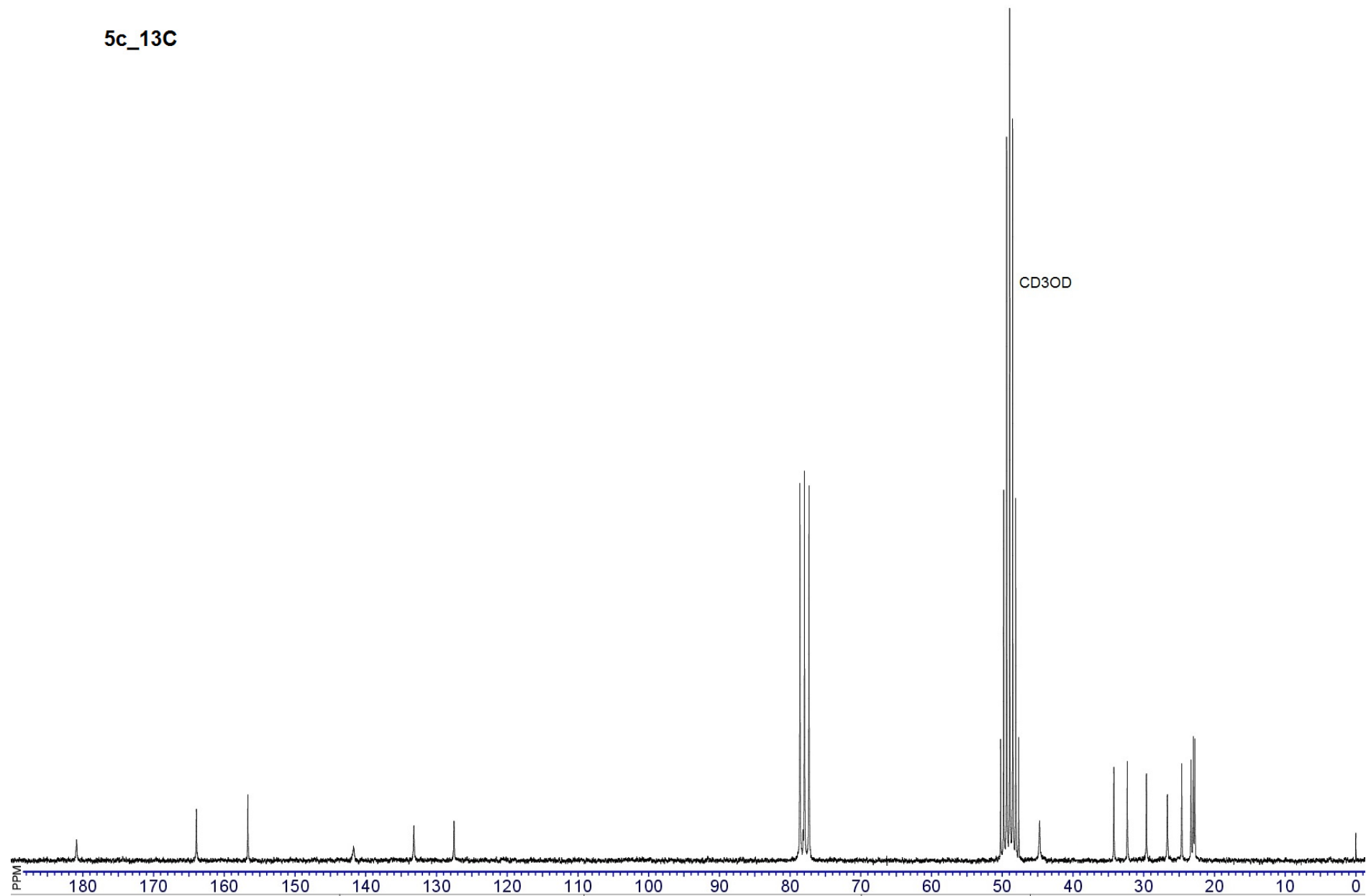

5d

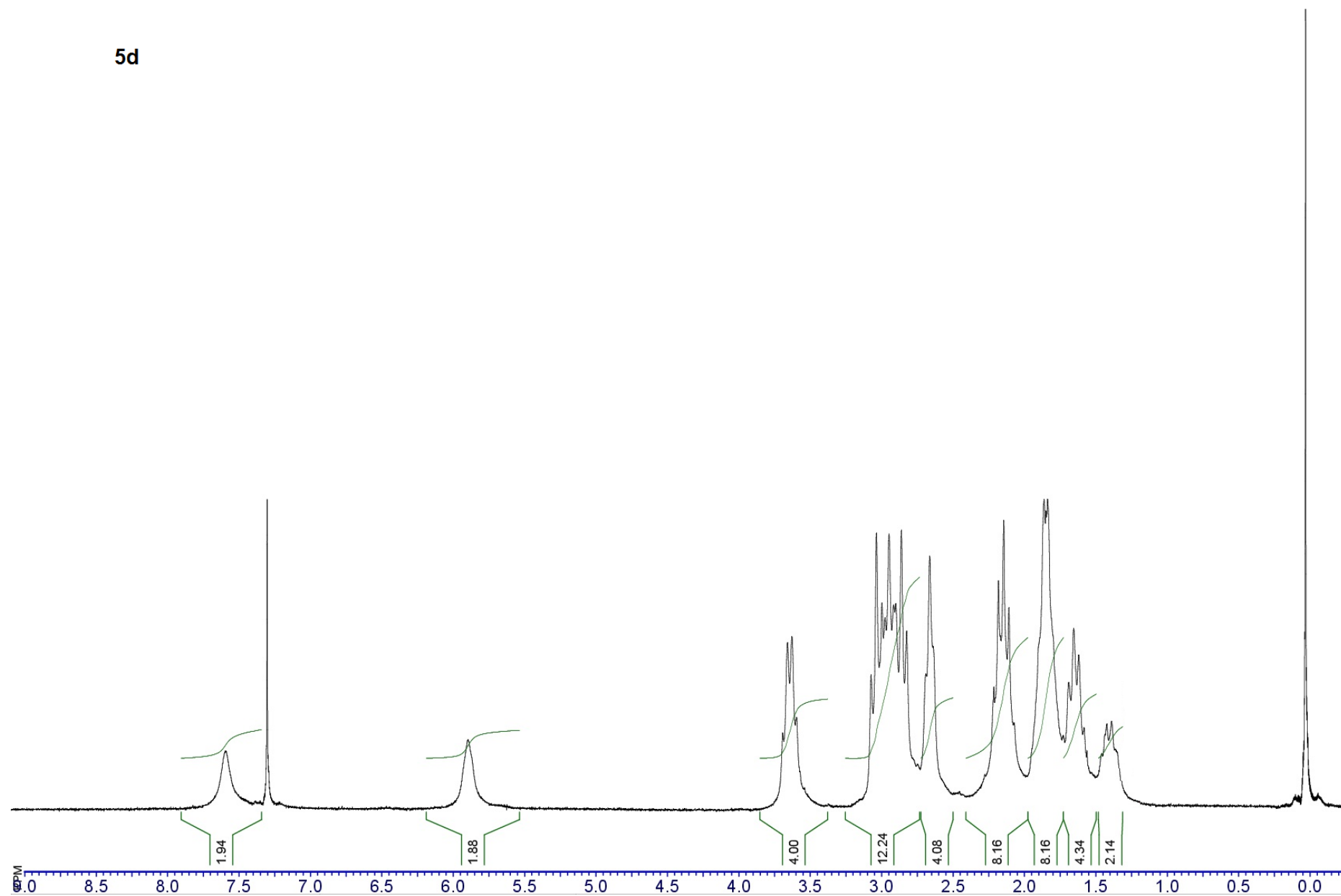

5d\_13C

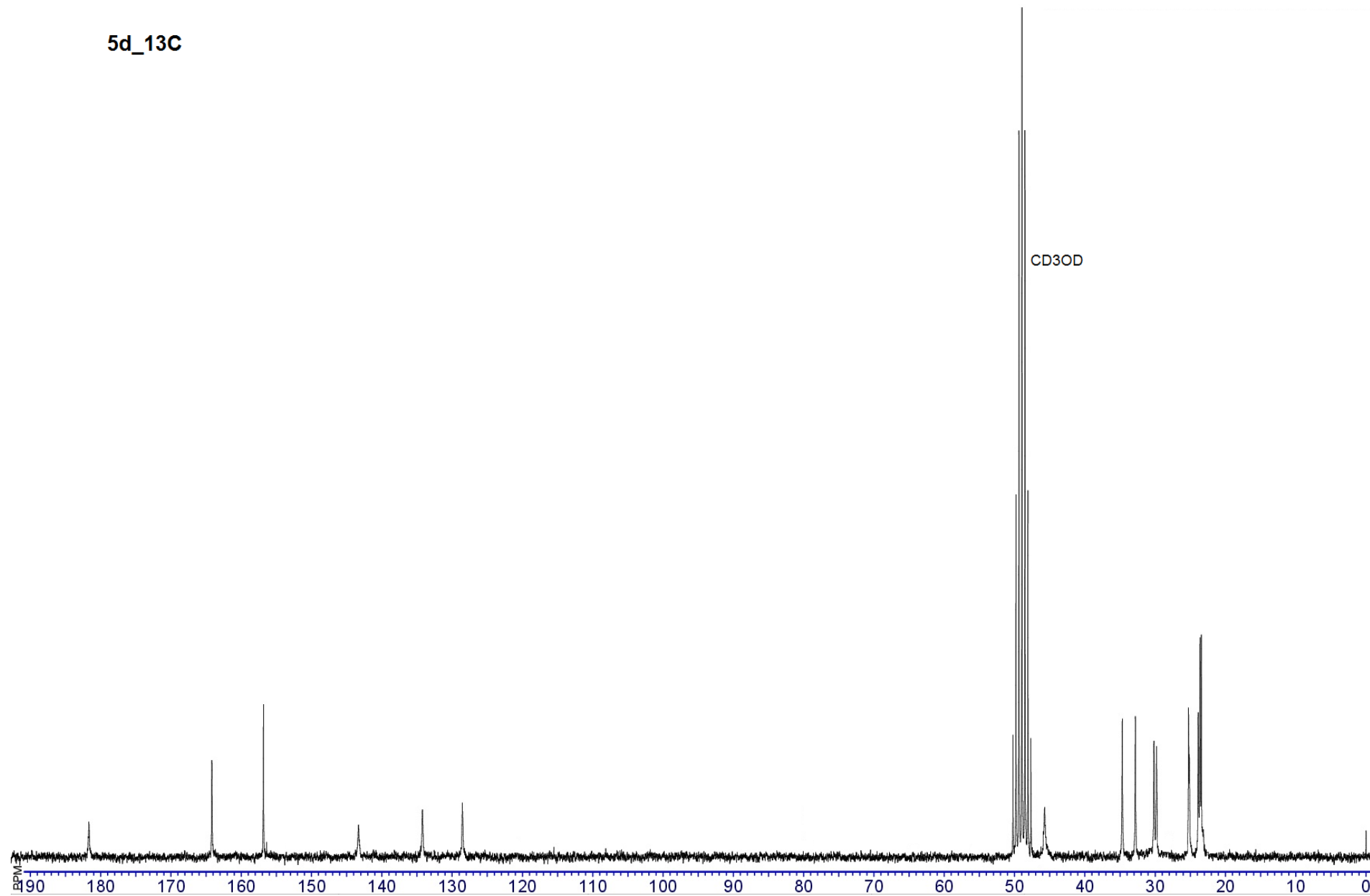

5e

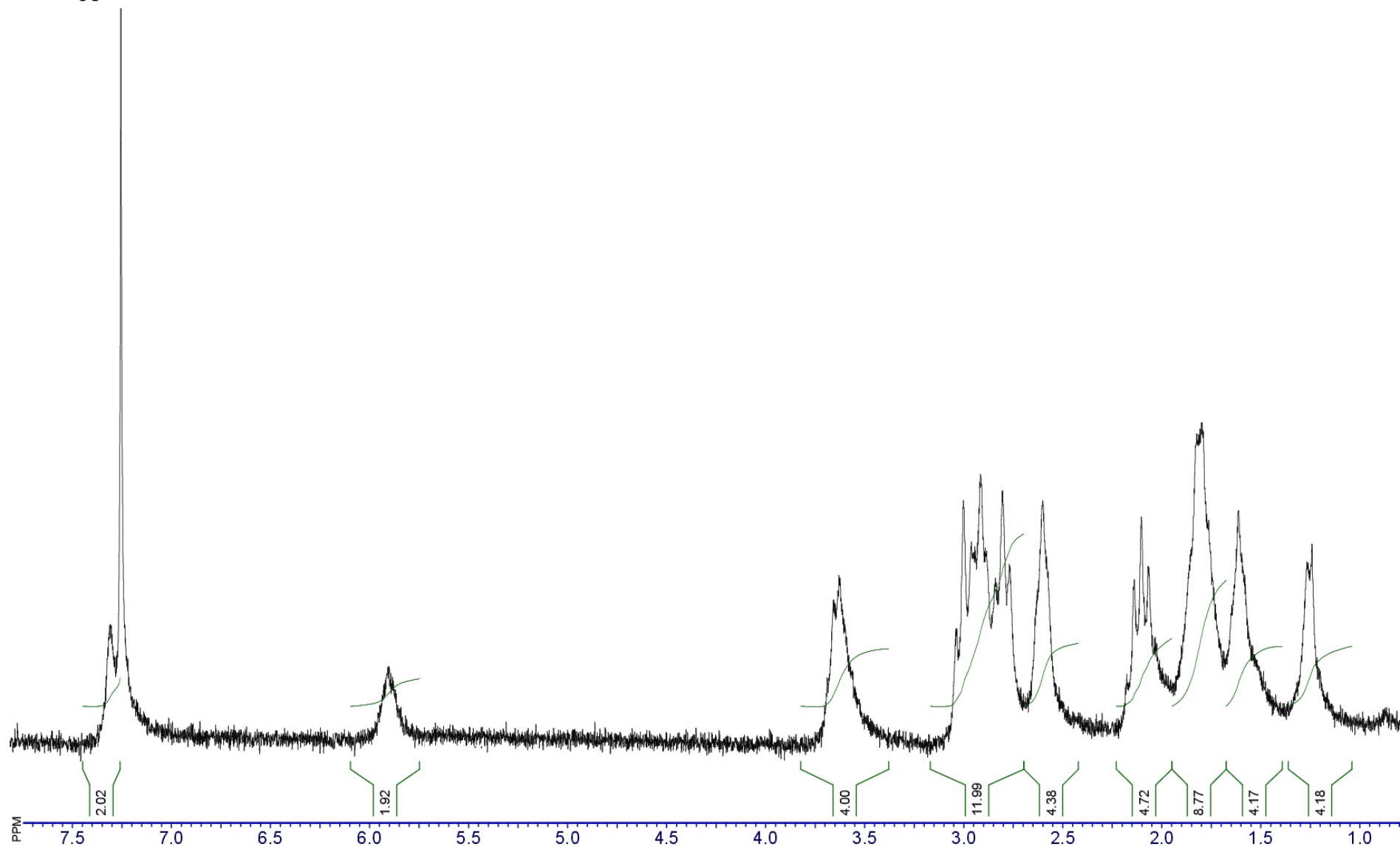

5e\_13C

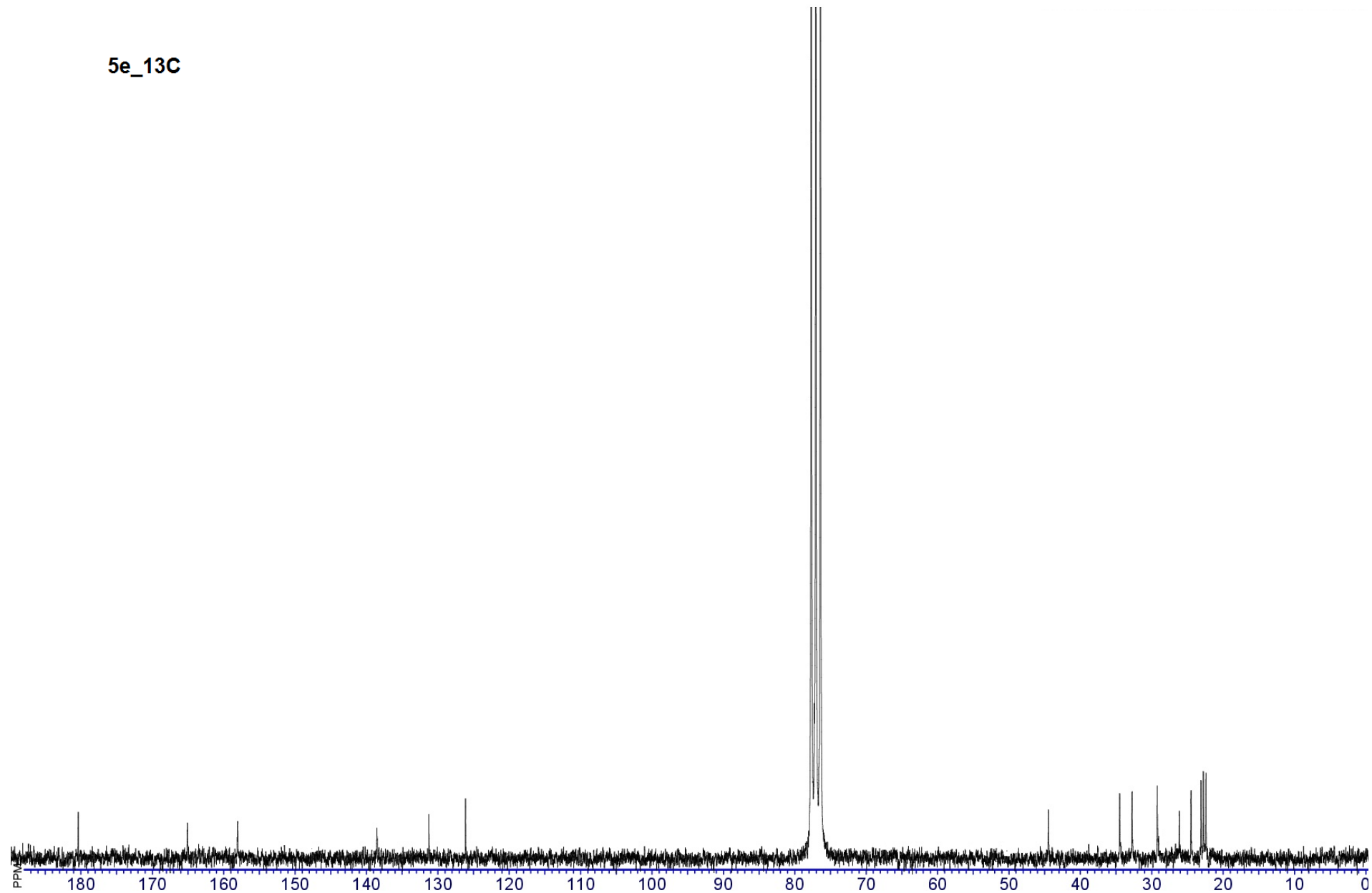

5f

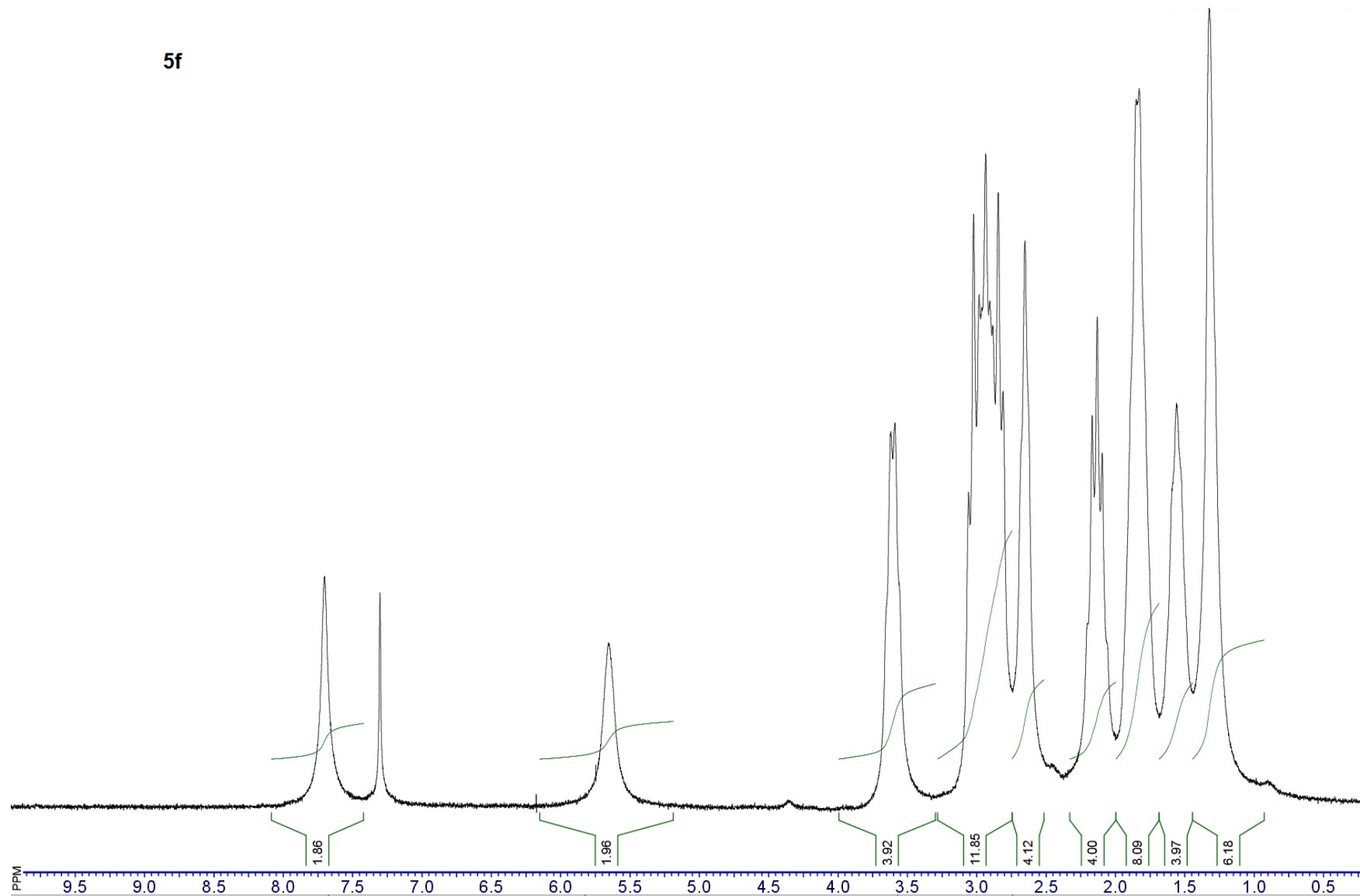

5f\_13C

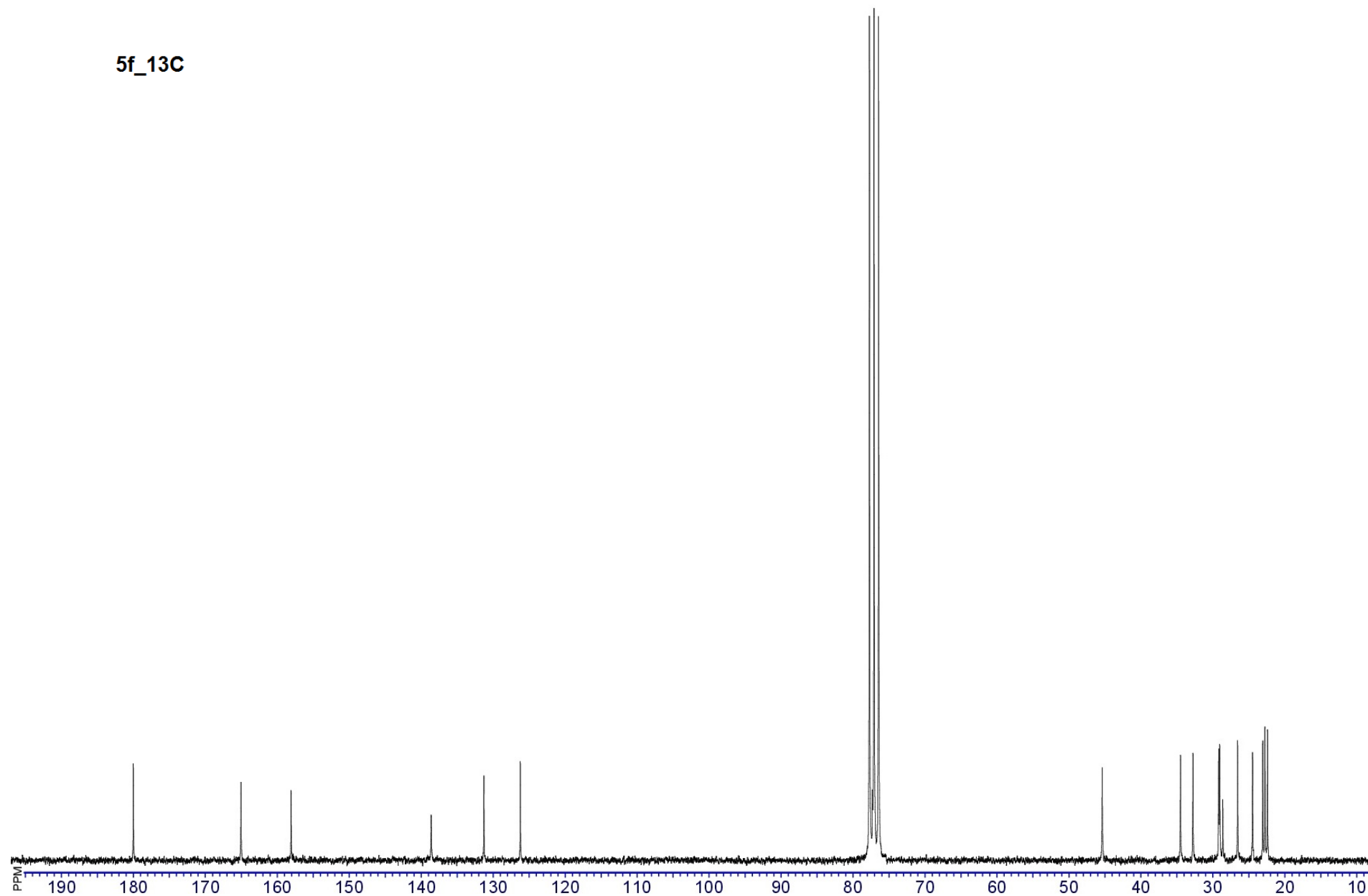

5g

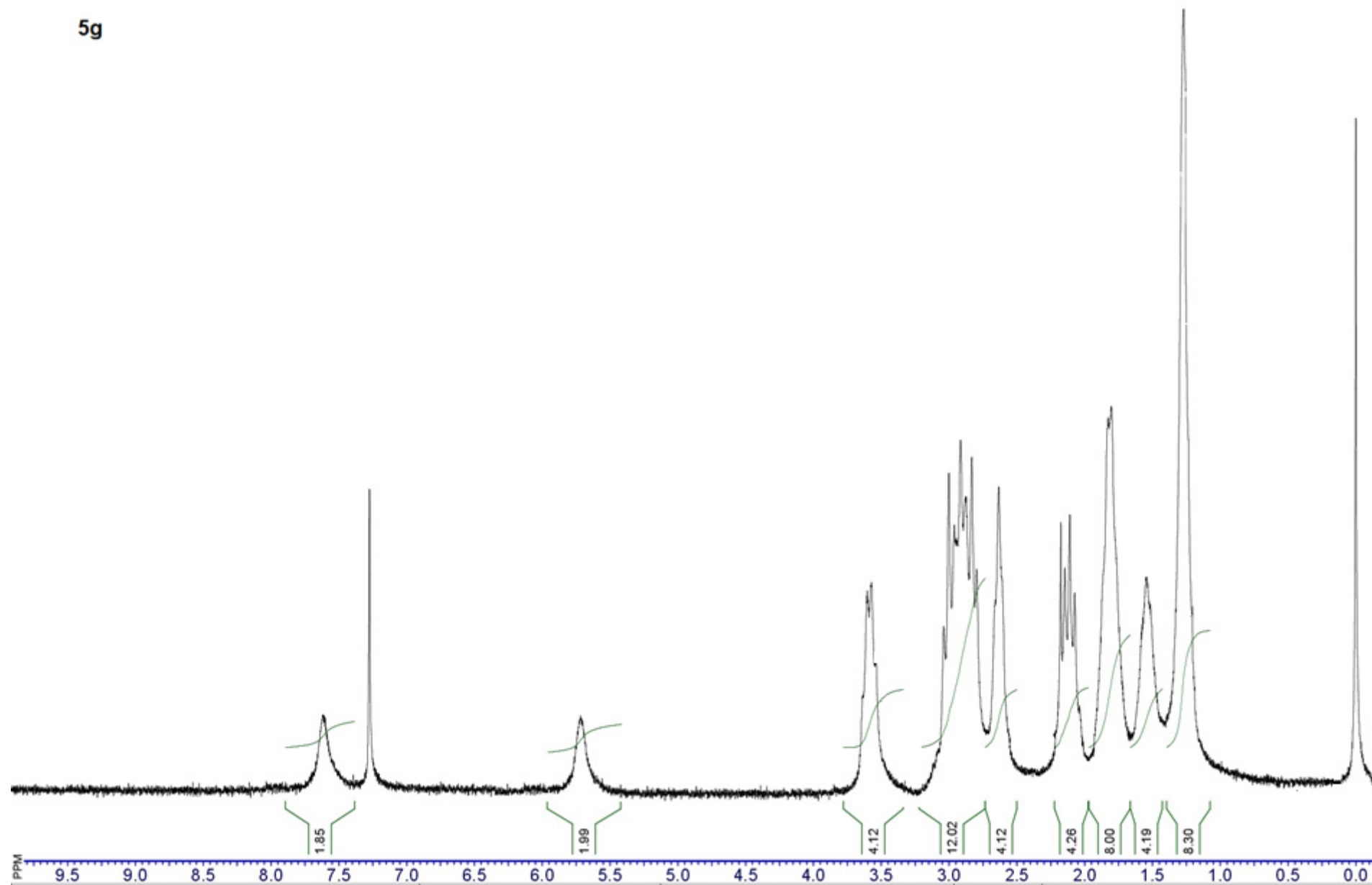

5g\_13C

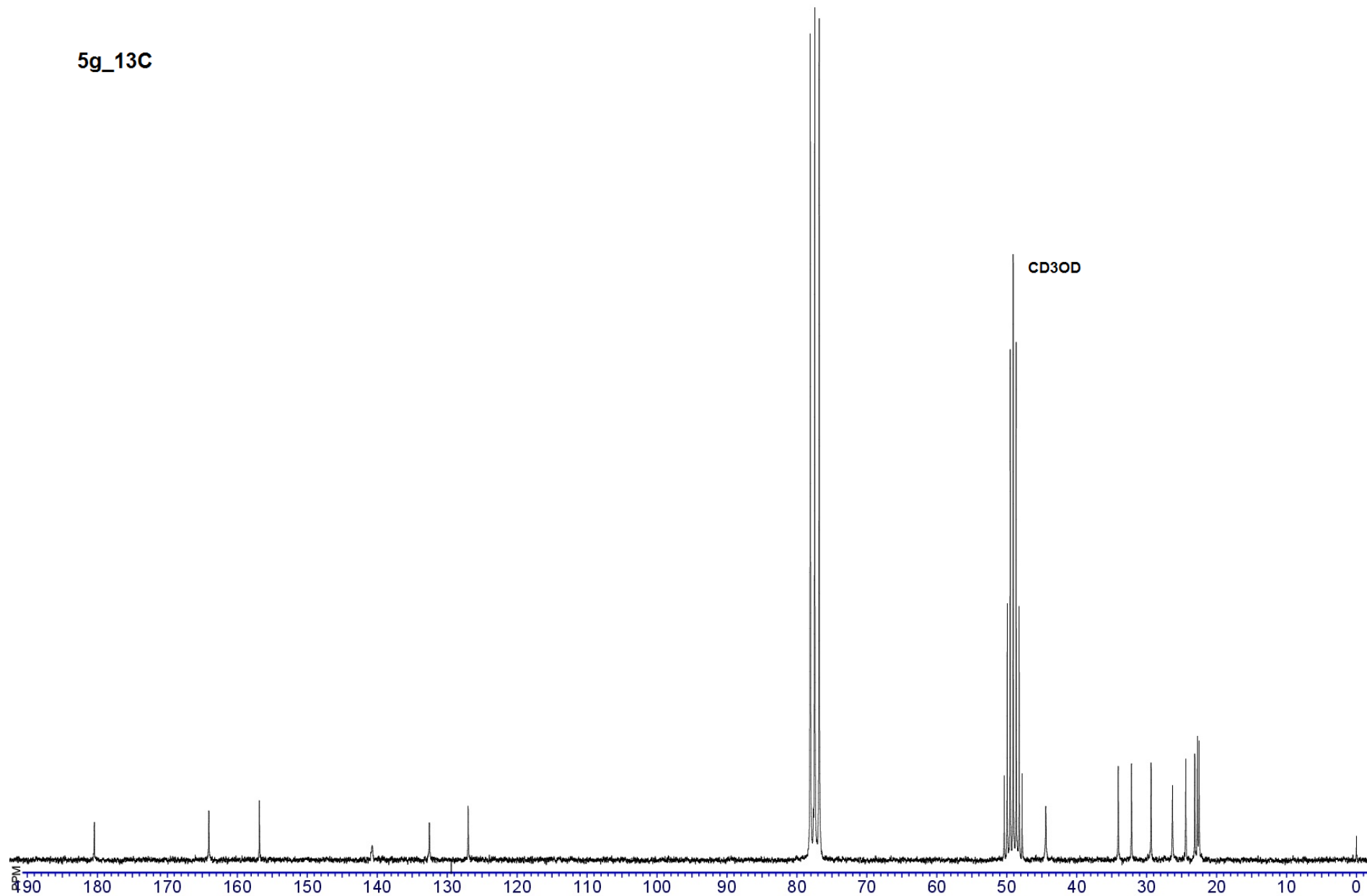

Supplement: Supplementary file 1 [file molecules-27-01060-s001.zip › FigS1-S23-Supplementary_spectra.pdf]
